# Comparable Metabolic and Histopathological Observations of Enzymatic and Non-Enzymatic Thai Shallot Extracts in High-Fat Diet-Induced Obese Mice

**DOI:** 10.3390/biology15130995

**Published:** 2026-06-24

**Authors:** Artorn Anuduang, Jiraporn Laoung-on, Oranit Kraseasintra, Somdet Srichairatanakool, Kittipan Rerkasem, Sakaewan Ounjaijean, Kongsak Boonyapranai

**Affiliations:** 1 Research Institute for Health Sciences, Chiang Mai University, Chiang Mai 50200, Thailand; artorn.a@cmu.ac.th (A.A.); jiraporn.l@cmu.ac.th (J.L.-o.); sakaewan.o@cmu.ac.th (S.O.); 2 Office of Research Administration, Chiang Mai University, Chiang Mai 50200, Thailand; orkraseasintra@gmail.com; 3 Department of Biochemistry, Faculty of Medicine, Chiang Mai University, Chiang Mai 50200, Thailand; somdet.s@cmu.ac.th; 4 Environmental—Occupational Health Sciences and Non Communicable Diseases Center of Excellence, 13 Research Institute for Health Sciences, Chiang Mai University, Chiang Mai 50200, Thailand

**Keywords:** Thai shallot extract, high-fat diet, insulin resistance, quercetin, oxidative stress, NAFLD

## Abstract

Thai shallot (*Allium cepa* L. var. *aggregatum*) is rich in quercetin glycosides, which can be enzymatically hydrolyzed into aglycone forms with potentially higher bioavailability. However, whether this structural modification enhances metabolic efficacy in vivo remains unclear. This study aimed to compare the metabolic and histopathological effects of enzymatic (ESE) and non-enzymatic (NES) Thai shallot extracts in a high-fat diet (HFD)-induced obese mouse model. Male C57BL/6 mice were fed HFD for 12 weeks to induce obesity, followed by a 12-week treatment with NES or ESE (1000 and 2000 mg/kg/day). Metabolic parameters, lipid profiles, oxidative stress markers, hepatic enzyme activities, and histopathological changes were evaluated. Enzymatic hydrolysis significantly increased the proportion of quercetin aglycone without altering total quercetin content. Both NES and ESE improved fasting glucose, insulin resistance, lipid profiles, and oxidative stress markers compared with HFD controls. Histological examination showed attenuation of hepatic steatosis and preservation of tissue architecture in treated groups. However, no consistent superiority of ESE over NES was observed across metabolic or histopathological outcomes. Despite substantial modification of flavonoid composition, enzymatic processing did not enhance the measured metabolic efficacy of Thai shallot extract under the conditions tested. Because circulating quercetin and metabolite levels were not assessed, this finding should be interpreted as comparable metabolic efficacy rather than evidence of equivalent bioavailability. These findings suggest that factors beyond aglycone content may play a key role in determining biological activity, with implications for the development and cost-effectiveness of functional food products.

## Simple Summary

Obesity and related conditions, such as high blood sugar and abnormal blood lipids, are major global health problems. Thai shallots contain natural compounds that may help improve these conditions. This study compared two types of shallot extracts prepared using different methods: a natural process and an enzyme-treated process that changes the form of a key compound called quercetin. Although the enzyme-treated extract contained more of the form that is believed to be more easily absorbed, both extracts showed similar beneficial effects in mice fed a high-fat diet. They helped lower blood sugar, improve blood lipid levels, reduce cell damage caused by oxidative stress, and decrease fat accumulation in the body and liver. No harmful effects on major organs were observed. These findings suggest that both extracts may be useful for developing affordable, health-promoting food products.

## 1. Introduction

Obesity and its associated metabolic disorders, including type 2 diabetes mellitus (T2DM), dyslipidemia, and insulin resistance, represent major global health challenges with increasing prevalence worldwide. These conditions are strongly linked to excessive consumption of high-fat diets (HFDs), which promote adiposity, disrupt glucose homeostasis, and impair lipid metabolism. In addition to metabolic imbalance, HFD-induced obesity is closely associated with oxidative stress and chronic low-grade inflammation, both of which play critical roles in the progression of metabolic diseases and organ dysfunction [1,2]. Given the limitations and side effects of current pharmacological interventions, there is growing interest in natural bioactive compounds as alternative or complementary strategies for the prevention and management of metabolic disorders.

Among dietary phytochemicals, flavonoids, particularly quercetin, have received considerable attention due to their antioxidant, anti-inflammatory, and metabolic regulatory properties. Quercetin has been shown to improve insulin sensitivity, reduce lipid accumulation, and modulate key metabolic pathways such as AMP-activated protein kinase (AMPK) and lipid homeostasis [3,4]. Notably, onions and shallots (*Allium cepa* L. and related species) are among the richest dietary sources of quercetin and other flavonoids, containing significantly higher levels than many common fruits and vegetables [5]. Accumulating evidence from both experimental and clinical studies suggests that *Allium*-derived compounds may exert beneficial effects on obesity, dyslipidemia, and glucose metabolism [6,7].

Thai shallot (*Allium cepa* L. var. *aggregatum ‘Chiang Mai’*), a widely consumed ingredient in Southeast Asia, has emerged as a promising functional food due to its rich phytochemical composition and biological activities. Previous studies by our research group have demonstrated that Thai shallot extracts possess potent antioxidant and anti-inflammatory properties in cellular models, including the ability to reduce intracellular reactive oxygen species (ROS), lipid peroxidation, and pro-inflammatory cytokine production [8]. In addition, in vivo studies have shown that shallot-based formulations can improve glycemic control, lipid profiles, and oxidative stress markers in diabetic animal models, supporting their potential role in metabolic disease management [9]. Collectively, these findings highlight the therapeutic potential of Thai shallot as a functional ingredient targeting oxidative stress and metabolic dysfunction.

Despite these promising effects, the biological activity of flavonoids is strongly influenced by their chemical structure and bioavailability. In plant sources, quercetin is predominantly present in glycoside forms, which require enzymatic hydrolysis prior to absorption, whereas aglycone forms are generally considered more readily absorbable in the gastrointestinal tract [10]. Consequently, enzymatic processing has been proposed as a strategy to enhance the bioavailability and efficacy of flavonoid-rich extracts by converting glycosides into aglycones. However, the in vivo relevance of this approach remains uncertain, as flavonoid metabolism involves complex interactions with intestinal enzymes, microbiota, and phase II conjugation pathways, which may influence systemic exposure and biological activity [11,12].

To date, limited studies have directly compared the metabolic effects of enzymatically modified and non-modified shallot extracts in diet-induced obesity models. Furthermore, most existing studies have focused primarily on biochemical outcomes, with relatively little attention given to histopathological changes in key metabolic organs. We hypothesized that enzymatic hydrolysis of Thai shallot extract would increase the proportion of quercetin aglycone, thereby enhancing its metabolic and histopathological benefits compared with the non-enzymatic extract in HFD-induced obese mice. Therefore, the present study aimed to compare the metabolic and histopathological effects of enzymatic and non-enzymatic Thai shallot extracts in a high-fat diet-induced obese mouse model. The novelty of this study lies in the direct comparison of these two extract types using integrated phytochemical, metabolic, oxidative stress, and histopathological analyses, thereby clarifying whether flavonoid structural modification translates into improved biological efficacy in vivo.

## 2. Materials and Methods

### 2.1. Materials

Bulbs of *Allium cepa* L. var. *aggregatum ‘Chiang Mai’*, cultivated without pesticides, were sourced from a local farm in Chiang Mai, Thailand (The geographical coordinates 18°38′39.9″ N, 98°49′34.3″ E). β-Glucosidase from almonds (Merck, Darmstadt, Germany), Lipid profile reagents were purchased from i-Med Laboratory Co., Ltd. (Bangkok, Thailand), automated clinical chemistry analyzer (Randox Laboratories, Crumlin, UK).

### 2.2. Sample Preparations

#### 2.2.1. Preparation of Shallot Extract

Fresh shallot extract was prepared according to the method of Ounjaijean and Somsak [13]. Shallots were peeled, and only the fleshy bulbs were used for extraction. The shallot juice was obtained using a juicer (Philips, Amsterdam, The Netherlands). After that, the extract was subjected to a drying process using a freeze dryer (Christ: Alpha 1–4 LSC plus, Harz, Germany). The resulting Thai shallot extract powder (TSE) was stored at −20 °C until further analysis.

#### 2.2.2. Enzymatic Hydrolysis of Shallot Extract

The method for preparing hydrolyzed shallot extract involves hydrolyzing 5 g of Thai shallot extract (TSE) with 0.05% β-Glucosidase (Sigma-Aldrich, Cat. No. G4511, St. Louis, MO, USA) in 0.1 mM phosphate buffer (pH 5.0), maintaining a TSE to phosphate buffer ratio of 1:10 (*w*/*v*). The mixture was incubated at 45 °C for 1 h, followed by heat treatment at 95 °C for 5 min in a water bath to deactivate the enzymes. After hydrolysis, the collected mixture was freeze-dried using a freeze dryer (Christ Alpha 1–4 LSC plus, Germany). The resulting enzymatic shallot extract powder (ESE) was stored at −20 °C for subsequent analysis. Non-enzymatic shallot extract (NES) was prepared similarly, but without the addition of β-glucosidase.

#### 2.2.3. Determination of Quercetin Aglycone and Glycosides

The quantification of quercetin and its glycosides in shallot extracts was performed using the method of Laoung-on et al. [8]. The analysis was conducted using a Shimadzu LC-20A HPLC system (Shimadzu Corporation, LC-20A, Kyoto, Japan) equipped with an autosampler, quaternary pump, and diode-array detector (DAD), with acquisition and control via LabSolutions software (version 5.82; Shimadzu Corporation, Kyoto, Japan). Separation was achieved on an Inertsil^®^ ODS-3 column (250 mm × 4.1 mm i.d., 5 μm pore size) maintained at 25 °C. The mobile phase consisted of solvent A (5% formic acid in water, *v*/*v*) and solvent B (methanol), with the following gradient: 0–5 min, 100:0 (A:B); 15–20 min, 85:15; 25–40 min, 40:60; 41–45 min, 0:100 (washing step); and 46–51 min, 100:0 (conditioning step). The flow rate was 1.0 mL/min. Detection was performed using diode-array scanning from 200 to 600 nm, with the wavelength for flavonol quantification set at 360 nm, a scan rate of 1 Hz, and a bandwidth of 2 nm. Peaks were identified by comparing retention times and UV–Vis spectra with those of authentic standards, and all quercetin derivatives were quantified using a calibration curve of quercetin-3-O-glucoside. Representative HPLC chromatograms of the non-enzymatic and enzymatic shallot extracts have been added to the Supplementary Materials as Supplementary Figure S1.

### 2.3. Animals

Male C57BL/6Mlac mice (8 weeks old, 15–25 g) were obtained from the National Laboratory Animal Center, Mahidol University, Salaya, Nakhon Pathom, Thailand. All mice were acclimatized for one week before the experimental procedures and housed under controlled temperature conditions (25 ± 1 °C) with a 12:12 h light/dark cycle and ad libitum access to food and water. All mice were fasted for 6 h before testing procedures. All experimental procedures were conducted in accordance with the Ethical Principles and Guidelines for the Use of Animals for Scientific Purposes of the National Research Council of Thailand and were approved by the Animal Ethics Committee, Faculty of Medicine, Chiang Mai University, Thailand (Protocol No. 38/2559: approval date: 15 December 2016). The animal experiments were conducted during the period covered by this approved protocol, and the resulting data have not been previously published.

### 2.4. Experimental Design, Diet Regimens, and Treatment Groups

The study consisted of two main phases: (1) induction of obesity and insulin resistance, and (2) treatment intervention.

For the normal control group, mice (*n* = 8) were fed a standard normal chow diet (NCD; 20% kcal fat, 52% kcal carbohydrates, 28% kcal protein; CP082) for 12 weeks and continued on the same diet throughout the experimental period. During the treatment phase, these mice received daily oral gavage of deionized water (2 mL/kg body weight) and served as the NCD control group (NCD-DW).

For the induction phase, mice (*n* = 40) were fed a high-fat diet (HFD; 52% kcal fat, 28% kcal carbohydrates, 20% kcal protein; SK019) for 12 weeks to induce obesity and insulin resistance. Body weight was monitored throughout the 12-week HFD feeding period. At the end of the induction phase, fasting glucose, insulin, and lipid profiles were measured, and HOMA-IR was calculated to confirm the development of obesity and insulin resistance.

The HFD-fed mice were then randomly assigned into five groups (*n* = 8 per group) for the treatment phase (12 weeks), while continuing on the HFD. Treatments were administered once daily via oral gavage as follows:

HFD-DW: High-fat diet + deionized water (2 mL/kg/day).

HFD-NES1: High-fat diet + non-enzymatic shallot extract (NES, 1000 mg/kg/day).

HFD-NES2: High-fat diet + non-enzymatic shallot extract (NES, 2000 mg/kg/day).

HFD-ESE1: High-fat diet + enzymatic shallot extract (ESE, 1000 mg/kg/day).

HFD-ESE2: High-fat diet + enzymatic shallot extract (ESE, 2000 mg/kg/day).

The selected doses were based on our previous in vivo study using Thai shallot extract in animal models [9], which demonstrated beneficial effects on glycemic control, lipid profiles, and oxidative stress markers. These doses were not based on allometric scaling from typical human dietary intake and should be considered pharmacological experimental doses rather than physiological dietary exposure levels. Throughout the experimental period, body weight and food intake were recorded regularly. Blood samples were collected at monthly intervals via the tail vein for biochemical analyses, including glucose, total triglycerides (TG), total cholesterol (TC), HDL-cholesterol, LDL-cholesterol, and oxidized LDL, using an automated clinical chemistry analyzer (Randox Laboratories, UK).

At the end of the study, mice were euthanized by overdose of thiopental sodium (250 mg/kg, i.p.). Major organs, including the liver, kidneys, heart, spleen, pancreas, and visceral white adipose tissue, were excised and weighed. For histopathological evaluation, tissue sections were coded and analyzed by an investigator blinded to the treatment groups. To ensure consistency among samples, all sections were examined using a standardized protocol in which multiple representative microscopic fields were systematically assessed across each tissue section. Visceral adiposity was expressed as the adiposity index, calculated as visceral white adipose tissue weight divided by final body weight × 100 (%WAT/BW).

### 2.5. Assessment of Insulin Resistance

Mice were fasted for 6 h prior to blood collection from the tail vein. The collected blood was centrifuged at 3000 rpm for 15 min, and the plasma was separated. Plasma insulin levels were measured using a Mouse Insulin ELISA kit (Sigma, St. Louis, MO, USA). Another portion of the plasma was used to determine fasting blood glucose levels using the O-toluidine assay method.

Insulin resistance was assessed based on the insulin resistance index [The homeostasis model assessment of insulin resistance (HOMA-IR)], calculated using the following equation:

HOMA-IR = [fasting insulin (μU/mL) × fasting glucose (mmol/L)]/22.5

### 2.6. Assessment of Lipid Peroxidation and Antioxidant Levels

Oxidative stress was evaluated by measuring markers of lipid peroxidation and antioxidant capacity in plasma and liver tissue. Lipid peroxidation levels were determined using the thiobarbituric acid reactive substances (TBARS) assay, which quantifies malondialdehyde (MDA) as an end product of lipid peroxidation. TBARS levels were measured in both plasma and liver tissue samples. Total antioxidant capacity in plasma was assessed using the ABTS radical cation decolorization assay. In addition, total glutathione levels in liver tissue were determined using a commercial glutathione assay kit (Sigma-Aldrich, St. Louis, MO, USA), according to the manufacturer’s instructions.

### 2.7. Statistical Analysis

All data are presented as mean ± standard deviation (SD). Statistical analyses were performed using STATA software (version 13.0; StataCorp, College Station, TX, USA). Sample sizes are indicated in the corresponding figure and table legends. Data distribution and homogeneity of variance were assessed using the Shapiro–Wilk test and Levene’s test, respectively. For comparisons between two independent groups, including validation of the HFD-induced obese phenotype between the NCD and HFD groups, an unpaired Student’s *t*-test was used. Comparisons among multiple groups were performed using one-way analysis of variance (ANOVA), followed by Tukey’s post hoc test for multiple comparisons. For parameters measured over time, including body weight and metabolic markers, two-way repeated-measures ANOVA was applied to assess group, time, and group × time interaction effects, followed by Tukey’s post hoc analysis where appropriate. Exact *p*-values were reported where applicable, and *p* < 0.05 was considered statistically significant.

## 3. Results

### 3.1. Phytochemical Characterization of Enzymatic and Non-Enzymatic Shallot Extracts

The phytochemical composition of quercetin and its derivatives in non-enzymatic (NES) and enzymatic (ESE) shallot extracts is summarized in Table 1. The total quercetin content was comparable between the two extracts, with NES and ESE containing 8.61 ± 0.49 and 9.07 ± 0.11 mg/g extract, respectively, indicating that enzymatic processing did not substantially alter the overall quercetin yield. In contrast, a marked difference was observed in the distribution of quercetin forms. NES was predominantly composed of quercetin glycosides, accounting for over 90% of total quercetin derivatives, with quercetin-4′-glucoside and quercetin-3,4′-diglucoside as the major constituents. In comparison, ESE exhibited a substantial increase in the proportion of quercetin aglycone (52.70 ± 2.46%), accompanied by a corresponding reduction in total glycosides (47.30 ± 1.72%). This shift was consistently observed across all glycoside subtypes, which were markedly decreased in ESE relative to NES.

These results indicate that enzymatic treatment effectively hydrolyzed quercetin glycosides into their aglycone form, leading to a significant alteration in the phytochemical profile of the extract without affecting total quercetin content. Given that quercetin aglycone has been reported to exhibit higher bioavailability compared with its glycoside counterparts, this compositional difference may have important implications for the biological activity of the extracts.

### 3.2. Validation of HFD-Induced Obese Phenotype

**Figure 1:**
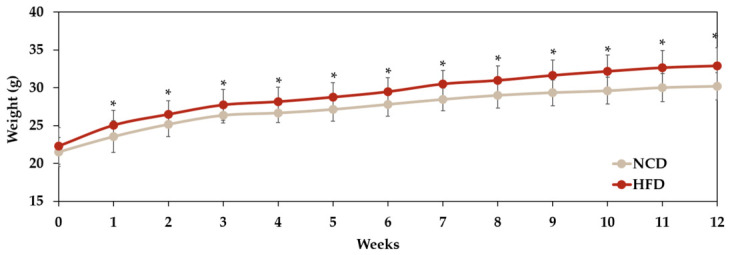
**Time-course of body weight changes during high-fat diet (HFD) induction.** Body weight changes in mice fed a normal chow diet (NCD, *n* = 8) or a high-fat diet (HFD, *n* = 40) over a 12-week induction period. Data are presented as mean ± SD. Statistical analysis was performed using two-way repeated measures ANOVA, followed by post hoc comparisons between NCD and HFD groups at each time point. * *p* < 0.05 vs. NCD at the corresponding time point.

**Figure 2:**
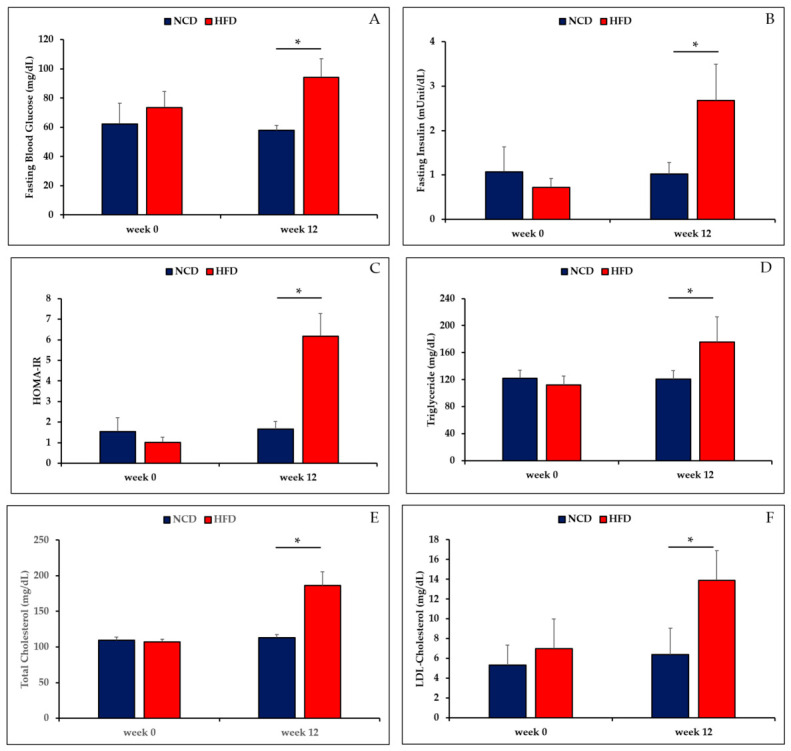
**Metabolic validation of high-fat diet (HFD)-induced obese and insulin-resistant phenotype.** Fasting metabolic parameters measured at baseline (week 0) and after 12 weeks of dietary induction in mice fed a normal chow diet (NCD, *n* = 8) or a high-fat diet (HFD, *n* = 40). (**A**) Fasting blood glucose, (**B**) fasting insulin, (**C**) HOMA-IR, (**D**) triglycerides (TG), (**E**) total cholesterol (TC), and (**F**) LDL-cholesterol. Data are presented as mean ± SD. Statistical comparisons between NCD and HFD groups at each time point were performed using an unpaired Student’s *t*-test. * *p* < 0.05 vs. NCD at the corresponding time point.

After 12 weeks of dietary induction, mice fed a high-fat diet (HFD) exhibited a progressive increase in body weight compared with those fed a normal chow diet (NCD) (Figure 1). At baseline, body weight was comparable between groups (NCD: 21.5 ± 1.93 g vs. HFD: 22.3 ± 2.42 g). However, from week 1 onward, HFD-fed mice consistently showed higher body weight, reaching 32.88 ± 2.38 g at week 12 compared with 30.19 ± 1.82 g in the NCD group. Metabolic parameters further confirmed the development of an obese and insulin-resistant phenotype (Figure 2). At baseline, fasting blood glucose, insulin, and HOMA-IR were similar between groups. After 12 weeks, HFD-fed mice exhibited significantly elevated fasting blood glucose (94.22 ± 12.9 mg/dL vs. 73.6 ± 11.09 mg/dL in NCD), fasting insulin (2.68 ± 0.82 µU/mL vs. 0.72 ± 0.2 µU/mL), and HOMA-IR (6.18 ± 1.09 vs. 1.02 ± 0.25).

In addition, HFD feeding induced marked dyslipidemia. At week 12, triglyceride levels were increased in HFD-fed mice (175.33 ± 37.83 mg/dL) compared with the NCD group (112 ± 13.26 mg/dL). Similarly, total cholesterol was elevated (186.21 ± 19.53 mg/dL vs. 107.33 ± 3.51 mg/dL), as well as LDL-cholesterol (13.89 ± 2.98 mg/dL vs. 6.97 ± 3.02 mg/dL). Taken together, these results demonstrate that 12 weeks of HFD feeding successfully induced obesity, insulin resistance, and dyslipidemia in mice, confirming the establishment of a metabolically impaired obese model for subsequent intervention studies.

### 3.3. Effects of Shallot Extracts on Body Weight and Food Intake

**Figure 3:**
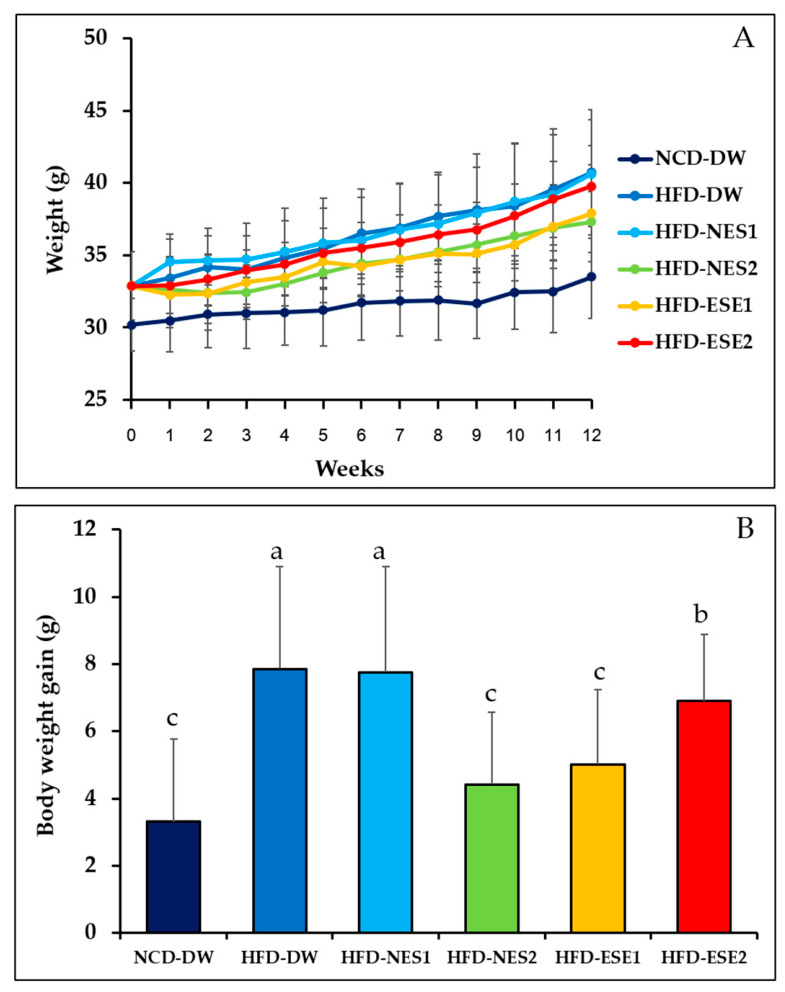
**Effects of enzymatic and non-enzymatic shallot extracts on body weight in HFD-fed mice.** (**A**) Body weight changes during the treatment period. (**B**) Total body weight gain over the treatment period. Data are presented as mean ± SD (*n* = 8 per group). Body weight changes over time were analyzed using two-way repeated-measures ANOVA to assess the effects of group, time, and group × time interaction, followed by Tukey’s post hoc test where appropriate. Total body weight gain was analyzed using one-way ANOVA followed by Tukey’s post hoc test. Groups not sharing the same letter are significantly different (*p* < 0.05).

Following the successful induction of obesity, the effects of enzymatic and non-enzymatic shallot extracts on body weight and food intake were evaluated during the 12-week treatment period in HFD-fed mice. During the treatment period, HFD-fed mice continued to exhibit a greater increase in body weight compared with the NCD group (Figure 3A). At the end of the study, body weight reached 40.73 ± 3.62 g in the HFD-DW group, compared with 33.50 ± 2.89 g in the NCD-DW group. Treatment with shallot extracts modulated body weight gain to varying degrees, with final body weights of 40.63 ± 4.43 g (HFD-NES1), 37.30 ± 2.10 g (HFD-NES2), 37.89 ± 3.36 g (HFD-ESE1), and 39.78 ± 2.78 g (HFD-ESE2). Consistent with these observations, total body weight gain was significantly higher in the HFD-DW group (7.85 ± 3.05 g) compared with the NCD-DW group (3.31 ± 2.45 g) (Figure 3B). Among treatment groups, HFD-NES1 exhibited a comparable weight gain to HFD-DW (7.75 ± 3.16 g), whereas HFD-NES2 (4.42 ± 2.14 g) and HFD-ESE1 (5.01 ± 2.22 g) showed reduced weight gain. The HFD-ESE2 group (6.90 ± 1.99 g) demonstrated an intermediate effect. Food intake did not differ significantly among experimental groups throughout the treatment period (Supplementary Figure S1), indicating that the observed differences in body weight were not attributable to changes in energy intake.

### 3.4. Effects of Shallot Extracts on Metabolic, Lipid, and Oxidative Parameters

**Figure 4:**
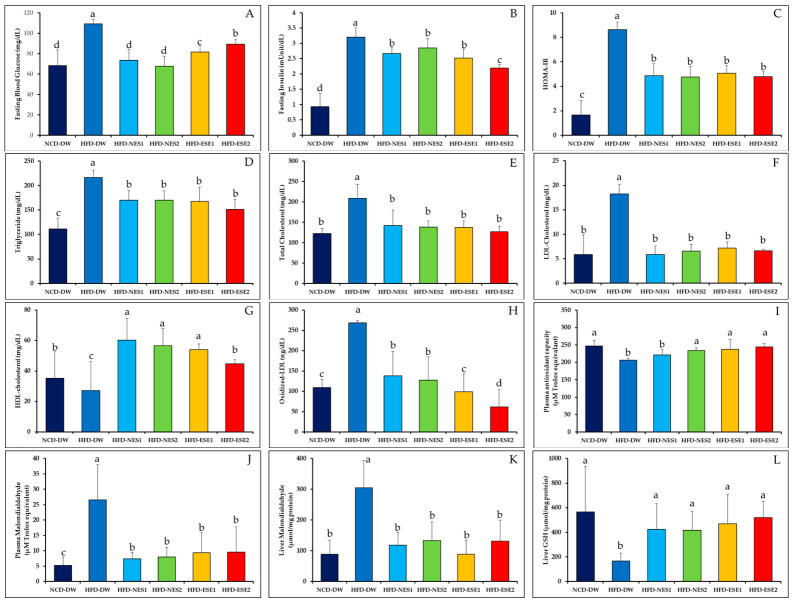
**Effects of enzymatic and non-enzymatic shallot extracts on glycemic control, insulin resistance, lipid profile, and antioxidant status in HFD-fed mice.** Fasting metabolic parameters measured after the 12-week treatment period in mice fed a normal chow diet or a high-fat diet with or without shallot extract treatment. (**A**) Fasting blood glucose, (**B**) fasting insulin, (**C**) HOMA-IR, (**D**) triglycerides, (**E**) total cholesterol, (**F**) LDL-cholesterol, (**G**) HDL-cholesterol, (**H**) oxidized-LDL, (**I**) Plasma TEAC, (**J**) Plasma MDA, (**K**) Liver MDA, and (**L**) Liver Glutathione. Data are presented as mean ± SD (*n* = 8 per group). Statistical analysis was performed using one-way ANOVA followed by Tukey’s post hoc test for multiple comparisons. Groups not sharing the same letter are significantly different (*p* < 0.05).

Treatment with shallot extracts improved multiple metabolic parameters in HFD-fed mice, including glycemic control, lipid profile, and oxidative stress status (Figure 4). Compared with the HFD-DW group, all treatment groups exhibited varying degrees of metabolic improvement, indicating a broad protective effect of shallot extracts against HFD-induced metabolic dysfunction.

Regarding glycemic control, HFD feeding significantly elevated fasting blood glucose, insulin, and HOMA-IR compared with the NCD group, confirming impaired glucose homeostasis (Figure 4A–C). Treatment with both non-enzymatic (NES) and enzymatic (ESE) extracts resulted in reductions in these parameters. Notably, HFD-NES2 restored fasting blood glucose to a level comparable to the NCD group, while all treatment groups significantly reduced insulin levels and HOMA-IR relative to HFD-DW. However, although improvements were evident, glycemic parameters in treated groups generally remained higher than those in the NCD group, indicating improvement in fasting indices of glycemic control and insulin resistance rather than definitive restoration of insulin sensitivity.

HFD feeding also induced marked dyslipidemia, as shown by increased triglycerides, total cholesterol, LDL-cholesterol, and oxidized LDL, along with reduced HDL-cholesterol (Figure 4D–H). Treatment with shallot extracts significantly improved lipid profiles, with reductions observed in triglycerides, total cholesterol, LDL-cholesterol, and oxidized LDL, alongside a marked increase in HDL-cholesterol. Importantly, HDL levels in all treated groups exceeded those of both HFD and NCD groups, suggesting a strong effect of shallot extracts on lipid modulation. Across lipid parameters, both NES and ESE exhibited comparable efficacy, with no consistent pattern indicating superior performance of enzymatic over non-enzymatic extracts.

In terms of oxidative stress, HFD-fed mice showed elevated plasma and liver MDA levels and reduced liver glutathione, indicating increased oxidative damage and compromised antioxidant defense (Figure 4I–L). Treatment with shallot extracts significantly reduced MDA levels in both plasma and liver and restored glutathione levels to values comparable to or higher than those of the NCD group. Plasma antioxidant capacity (TEAC) showed moderate improvement. These findings indicate that shallot extracts exert protective effects against oxidative stress, likely contributing to the observed improvements in metabolic parameters.

The overall pattern of metabolic improvement was further supported by the heatmap analysis (Table 2), which demonstrated consistent normalization of glycemic, lipid, and oxidative stress markers relative to the HFD control. Notably, the magnitude of improvement across parameters was largely similar between NES and ESE groups, suggesting that enzymatic processing did not confer a clear advantage over non-enzymatic extracts under the conditions tested. This comparable efficacy highlights the potential of both extract types as functional interventions for metabolic disorders.

### 3.5. Effects of Shallot Extracts on Organ Weights and Visceral Fat

The effects of shallot extracts on body weight, organ weights, and visceral adiposity are summarized in Table 3. HFD feeding resulted in a significant increase in body weight and adiposity compared with the NCD group, confirming the successful induction of obesity. In particular, white adipose tissue (WAT) weight was markedly elevated in HFD-fed mice (3.22 ± 0.79 g) compared with the NCD group (0.18 ± 0.03 g). Consistently, the percentage of WAT relative to body weight (%WAT/BW) was significantly increased in the HFD group (7.82 ± 1.44%) compared with the NCD group (0.53 ± 0.10%), further indicating excessive fat accumulation.

Treatment with both non-enzymatic (NES) and enzymatic (ESE) shallot extracts moderately attenuated visceral fat accumulation. This effect was reflected by reduced WAT weights in HFD-NES1, HFD-NES2, and HFD-ESE1 groups (approximately 2.77–2.84 g), with corresponding decreases in %WAT/BW (6.76–7.48%) compared with the HFD-DW group. However, these values remained substantially higher than those of the NCD group. The HFD-ESE2 group showed a less pronounced reduction in adiposity, with both WAT weight and %WAT/BW values comparable to those of the HFD control.

With respect to organ weights, HFD feeding did not markedly affect most organs, including the heart and spleen, which remained relatively stable across groups. Liver weight showed a slight decrease in HFD-fed mice compared with the NCD group and was further reduced in some treatment groups, particularly HFD-NES2. Kidney weights exhibited minor variations, with slight reductions observed in HFD-fed mice and partial normalization following treatment. Interestingly, spleen weight tended to increase in several treatment groups compared with the HFD control, suggesting a potential modulation of immune-related responses. In contrast, pancreas weight showed variable changes, with some treatment groups exhibiting reductions relative to the HFD group, although these changes were not consistent across treatments.

Overall, shallot extract treatment exerted a modest effect on organ weights but demonstrated a more consistent impact on reducing visceral adiposity, as supported by both absolute WAT weight and %WAT/BW. Importantly, no overt signs of organ toxicity were observed, as major organ weights remained within a relatively narrow range across groups.

### 3.6. Hepatotoxicity Assessment and Histopathological Analysis

**Figure 5:**
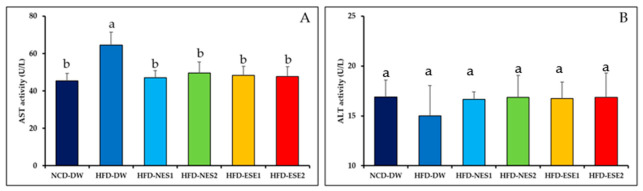
**Effects of enzymatic and non-enzymatic shallot extracts on hepatic enzyme markers in HFD-fed mice (*n* = 8 per group).** (**A**) Aspartate aminotransferase (AST) and (**B**) alanine aminotransferase (ALT) activities measured after the 12-week treatment period. Data are presented as mean ± SD. Statistical analysis was performed using one-way ANOVA followed by Tukey’s post hoc test. Groups not sharing the same letter are significantly different (*p* < 0.05).

Histopathological examination of major organs, including the liver, kidney, spleen, heart, and pancreas, was performed to further evaluate tissue-level alterations associated with HFD feeding and the effects of shallot extract treatment (Table 4). In the liver, HFD-fed mice exhibited pronounced structural alterations compared with the NCD group, characterized by features consistent with hepatic lipid accumulation and disrupted hepatocellular architecture. Histologically, these changes included mild to moderate microvesicular steatosis, hepatocellular enlargement, and flocculent cytoplasmic appearance, accompanied by alterations in both cytoplasmic and nuclear morphology. Multiple lipid droplets of varying sizes were observed within hepatocytes, indicative of non-alcoholic fatty liver disease (NAFLD)-like changes induced by HFD feeding. These observations are in line with the elevated AST levels observed in the HFD group (Figure 5), indicating hepatic stress. Treatment with both non-enzymatic (NES) and enzymatic (ESE) shallot extracts partially improved liver histology, with a more preserved cellular architecture and reduced apparent lipid accumulation. These improvements were reflected by decreased steatotic features and fewer lipid droplets compared with the HFD control. Consistent with these findings, AST activity was reduced in treated groups, alongside improvements in lipid and oxidative stress markers, suggesting attenuation of HFD-induced hepatic dysfunction.

Renal histology showed no obvious pathological abnormalities across groups (Table 4). Although minor structural variations were observed in HFD-fed mice, treatment groups displayed overall preserved renal architecture, indicating no adverse effects on kidney tissue. This observation is consistent with the relatively stable kidney weights reported in Table 1. In the spleen, HFD feeding appeared to alter tissue organization compared with the NCD group. Treatment with shallot extracts resulted in subtle changes in splenic morphology, which may reflect modulation of immune-related processes. This finding is in agreement with the slight increase in spleen weight observed in some treatment groups. Cardiac tissue showed no evident histopathological alterations across all experimental groups, indicating that neither HFD feeding nor shallot extract treatment induced structural damage in the heart. Similarly, pancreatic islet architecture was relatively preserved across groups, although minor variations were observed in HFD-fed mice and partially improved in treated groups. Overall, histopathological findings support the biochemical data, demonstrating that shallot extract treatment alleviated HFD-induced tissue alterations, particularly in the liver, without inducing observable tissue toxicity. These results are consistent with the improvements in metabolic, lipid, and oxidative stress parameters, further supporting the protective effects of shallot extracts.

## 4. Discussion

The present study demonstrates that both enzymatic and non-enzymatic Thai shallot extracts exert beneficial effects on metabolic dysfunction, lipid abnormalities, and oxidative stress in a high-fat diet (HFD)-induced obese mouse model. Despite a substantial shift in flavonoid composition following enzymatic hydrolysis, particularly the marked increase in quercetin aglycone content, no consistent superiority of enzymatic shallot extract (ESE) over non-enzymatic extract (NES) was observed across metabolic, biochemical, or histopathological outcomes. These findings provide important insights into the relationship between flavonoid structure and biological efficacy in vivo, suggesting that increased aglycone content alone may not be the primary determinant of metabolic benefit under the conditions tested.

The high-fat diet (HFD)-induced obese mouse model used in this study successfully reproduced key features of metabolic syndrome, including increased body weight, visceral adiposity, insulin resistance, dyslipidemia, and hepatic steatosis. Chronic consumption of HFD is known to promote excessive lipid accumulation in adipose tissue and liver, leading to adipocyte hypertrophy, ectopic fat deposition, and impaired metabolic homeostasis [14,15]. This metabolic overload is closely associated with the development of insulin resistance, driven by lipid-induced cellular stress, mitochondrial dysfunction, and low-grade inflammation [16]. In addition, HFD contributes to increased oxidative stress through enhanced production of reactive oxygen species, which further exacerbates metabolic dysfunction and tissue damage [17]. The liver is particularly vulnerable, as excess fatty acid influx promotes the development of non-alcoholic fatty liver disease (NAFLD), characterized by steatosis and hepatocellular injury [18]. The consistency of these pathological changes with previous reports supports the validity of the HFD model in mimicking human metabolic disorders and provides a relevant platform for evaluating the therapeutic potential of functional food interventions.

The metabolic improvements observed in both NES- and ESE-treated groups are consistent with previous reports demonstrating the anti-obesity, anti-hyperlipidemic, and insulin-sensitizing effects of quercetin and Allium-derived phytochemicals [19,20,21,22,23,24]. In the present study, treatment with shallot extracts led to reductions in fasting glucose, insulin levels, and HOMA-IR, indicating improvement in fasting glycemic parameters and fasting-derived indices of insulin resistance. Similarly, improvements in lipid profiles, including decreased triglycerides, total cholesterol, LDL-cholesterol, and oxidized LDL, along with increased HDL-cholesterol, suggest a broad modulatory effect on lipid metabolism. These effects are likely mediated, at least in part, by the antioxidant properties of quercetin and related flavonoids, which have been shown to regulate key metabolic pathways such as AMPK activation, lipid oxidation, and inflammatory signaling [3,4].

Oxidative stress is a central mechanism underlying HFD-induced metabolic dysfunction, contributing to insulin resistance, hepatic steatosis, and chronic inflammation. In this study, both NES and ESE significantly reduced lipid peroxidation, as evidenced by decreased plasma and liver MDA levels, and enhanced antioxidant defense through restoration of glutathione levels. These findings are in agreement with previous studies reporting the antioxidant and cytoprotective effects of quercetin and Allium extracts [25,26]. The attenuation of oxidative stress likely contributes to the observed improvements in metabolic parameters and supports the role of shallot-derived phytochemicals as functional agents in mitigating diet-induced metabolic disturbances.

The liver plays a central role in metabolic regulation and is particularly susceptible to lipid accumulation under HFD conditions. Histopathological analysis in this study revealed NAFLD-like features in HFD-fed mice, including microvesicular steatosis, hepatocellular enlargement, and the presence of lipid droplets of varying sizes [18]. These structural changes were accompanied by elevated AST levels, indicating hepatic stress. Treatment with shallot extracts partially improved liver histology, reducing steatotic features and preserving hepatocellular architecture, in agreement with the observed reduction in AST activity. These findings suggest a hepatoprotective effect of shallot extracts, likely mediated through combined antioxidant and lipid-lowering mechanisms. Similar protective effects of quercetin against hepatic steatosis have been reported in previous studies [27].

An important observation in this study is that both body weight gain and visceral adiposity were attenuated in extract-treated groups compared with the HFD control, although these effects did not reach statistical significance in all comparisons. HFD feeding significantly increased body weight relative to the NCD group, while mice receiving shallot extracts showed a lower extent of weight gain, indicating a modest mitigating effect. A similar trend was observed for white adipose tissue (WAT), where absolute fat mass tended to decrease in treated groups. However, no clear differences were observed in %WAT/BW between treated and untreated HFD groups, suggesting that the relative proportion of adiposity was not substantially altered. This indicates that the extracts may exert a general effect on overall weight gain and fat accumulation rather than selectively modifying fat distribution. Despite these modest changes, the consistent trends in adiposity, together with the pronounced improvements in glycemic control, lipid metabolism, and oxidative stress, suggest that metabolic benefits may occur independently of significant reductions in body weight or relative fat mass [16,28]. This further suggests that the primary effects of the extracts are metabolic rather than anti-obesity in nature.

A key finding of this study is the lack of a clear advantage of enzymatic hydrolysis in enhancing metabolic efficacy, despite significantly increasing the proportion of quercetin aglycone. This observation contrasts with the common assumption that aglycone forms exhibit superior bioavailability and biological activity compared with glycosides. Previous human studies have shown that quercetin glycosides can be efficiently absorbed and may demonstrate comparable or even higher bioavailability than the aglycone form, depending on the food matrix and intestinal processing [29,30]. Although aglycones are generally considered more readily absorbed in the small intestine [10], flavonoid metabolism in vivo is complex and involves intestinal hydrolysis, microbial transformation, and extensive phase II conjugation, including glucuronidation and sulfation [11,12]. Therefore, the comparable efficacy observed between NES and ESE may reflect the contribution of downstream metabolites rather than the parent compounds alone.

In addition, the relatively high doses used in this study may have minimized differences in bioavailability between the two extract types. Previous human studies have shown that plasma quercetin concentrations increase in a dose-dependent manner following supplementation, suggesting that absorption capacity may approach saturation at higher intake levels [31]. Furthermore, gut microbiota may reduce functional differences between enzymatic and non-enzymatic extracts because intestinal microorganisms can hydrolyze flavonoid glycosides and further metabolize quercetin into smaller bioactive phenolic compounds [32,33,34,35,36]. Collectively, these findings suggest that increasing aglycone content through enzymatic processing may not necessarily translate into enhanced biological efficacy in vivo, particularly under high-dose supplementation conditions.

From a translational perspective, these findings have important implications for the development of functional food products. Enzymatic hydrolysis adds complexity, cost, and processing requirements, and the absence of a clear efficacy advantage raises questions regarding its practical value. The comparable metabolic benefits observed with NES suggest that simpler extraction methods may be sufficient to achieve desired health effects, potentially improving the cost-effectiveness and scalability of shallot-based functional ingredients.

Based on the parameters evaluated in this study, including organ weights, hepatic enzyme activities, and histopathological examination, no overt signs of organ toxicity were observed following daily oral administration of shallot extracts for 12 weeks. However, this study was not designed as a formal toxicity assessment, and comprehensive toxicological endpoints were not evaluated. Therefore, the present findings should be interpreted as preliminary evidence of tolerability under the experimental conditions tested, rather than definitive evidence of long-term safety. Dedicated sub-chronic and chronic toxicity studies are required to fully establish the safety profile of Thai shallot extracts for functional food or nutraceutical applications.

Several limitations of this study should be acknowledged. First, although enzymatic hydrolysis markedly increased the proportion of quercetin aglycone in ESE, this study did not directly assess pharmacokinetic parameters, tissue distribution, or plasma quercetin metabolites. Therefore, whether ESE improved systemic quercetin exposure or bioavailability compared with NES remains unconfirmed. This limitation restricts the ability to directly link flavonoid structural modification with in vivo efficacy, and future studies using pharmacokinetic analysis and plasma or tissue metabolite profiling are needed to clarify this relationship. In addition, the present study focused on quercetin aglycone and selected quercetin glycosides as major flavonoid markers of Thai shallot extract. We acknowledge that the biological activity of the extract may also involve other flavonoids, organosulfur compounds, phenolic acids, and unidentified phytochemicals. Therefore, comprehensive chromatographic and metabolomic profiling should be performed in future studies to better characterize the full phytochemical composition and its relationship with biological activity.

Second, this study did not include a positive control group, such as metformin, a quercetin standard, or an established anti-obesity agent. Therefore, the relative efficacy of Thai shallot extracts compared with standard therapeutic or bioactive reference compounds could not be determined. Future studies should include appropriate positive controls to better benchmark the metabolic effects of enzymatic and non-enzymatic shallot extracts.

Third, dynamic assessments of glucose metabolism, such as oral glucose tolerance tests (OGTT) or insulin tolerance tests (ITT), were not performed. Therefore, the present findings should be interpreted as improvements in fasting glucose, fasting insulin, and HOMA-IR, rather than definitive evidence of restored whole-body glucose tolerance or insulin sensitivity.

Fourth, the mechanistic basis of the observed effects was not explored at the molecular level, including the evaluation of AMPK, PPARs, inflammatory cytokines, and lipid metabolism-related genes; therefore, the proposed mechanisms remain inferential. Future studies incorporating molecular analyses are required to clarify the pathways underlying the metabolic and antioxidant effects of Thai shallot extracts.

Fifth, histopathological evaluation was primarily descriptive and did not include quantitative scoring systems, which may limit the objectivity of tissue-level comparisons. Finally, the doses used in this study were relatively high and should be considered pharmacological experimental doses rather than physiological dietary exposure levels, as they were derived from prior rodent work and were not based on allometric scaling from typical human intake. This may limit direct translational applicability to human populations.

Taken together, this study demonstrates that Thai shallot extracts, regardless of enzymatic processing, provide significant metabolic and antioxidant benefits in a diet-induced obesity model. The lack of enhanced efficacy following enzymatic hydrolysis suggests that factors beyond aglycone content, including metabolic transformation and host–microbiota interactions, may play a dominant role in determining biological activity. These findings highlight the importance of evaluating functional food processing strategies in the context of whole-system physiology and support the potential use of non-enzymatic shallot extracts as cost-effective functional ingredients for metabolic health.

## 5. Conclusions

Thai shallot extracts, both enzymatic and non-enzymatic, improved metabolic parameters and lipid profiles and reduced oxidative stress markers in a high-fat diet-induced obese mouse model. Although enzymatic hydrolysis significantly increased quercetin aglycone content, it did not result in superior measured metabolic or histopathological outcomes compared with the non-enzymatic extract. Because circulating quercetin, quercetin metabolites, and tissue distribution were not assessed, this comparable efficacy should be interpreted as comparable biological outcomes under the present experimental conditions, rather than evidence of equivalent bioavailability or systemic exposure. These findings suggest that aglycone enrichment alone may not determine biological efficacy in vivo, and that factors such as metabolic transformation and gut microbiota may play important roles. From a practical standpoint, the comparable efficacy indicates that enzymatic processing may not be required, supporting simpler and more cost-effective production of shallot-based functional ingredients. However, the lack of pharmacokinetic and mechanistic data limits interpretation. Future studies should include pharmacokinetic analysis, mechanistic evaluation, and comprehensive chromatographic or metabolomic profiling to better clarify the relationship between phytochemical composition, bioavailability, and biological efficacy.

## Figures and Tables

**Table 1:** Quercetin composition of enzymatic and non-enzymatic shallot extracts.

Composition	NES	ESE
Total quercetin (mg/g extract)	8.61 ± 0.49	9.07 ± 0.11
Quercetin aglycone (%)	7.47 ± 0.34	52.70 ± 2.46
Quercetin glycosides (%)	92.53 ± 3.15	47.30 ± 1.72
- Quercetin-4′-glucoside (%)	38.91 ± 2.07	13.71 ± 0.93
- Quercetin-3,4′-diglucoside (%)	40.50 ± 0.69	28.27 ± 1.17
- Quercetin-3-glucoside (%)	2.71 ± 0.18	1.41 ± 0.20
- Other glycosides (%)	10.41 ± 3.21	3.91 ± 0.68

Data are presented as mean ± SD (*n* = 3). The relative composition of individual quercetin glycosides is expressed as a percentage of total quercetin derivatives.

**Table 2:** Heatmap representation of treatment effects on metabolic, lipid, and oxidative stress parameters relative to HFD control.

Group	Parameters	NCD-DW	HFD-DW	HFD-NES1	HFD-NES2	HFD-ESE1	HFD-ESE2	Beneficial Direction
glycemic control and insulin resistance	Fasting blood glucose	0.63	1.00	0.67	0.62	0.75	0.82	↓
	Fasting insulin	0.29	1.00	0.83	0.89	0.79	0.69	↓
	HOMA-IR	0.19	1.00	0.56	0.55	0.59	0.55	↓
lipid profile	Triglycerides	0.51	1.00	0.79	0.79	0.77	0.70	↓
	Total cholesterol	0.59	1.00	0.68	0.66	0.66	0.61	↓
	LDL-C	0.32	1.00	0.32	0.36	0.39	0.36	↓
	HDL-C	1.30	1.00	2.23	2.10	2.00	1.65	↑
	oxLDL	0.41	1.00	0.51	0.48	0.37	0.23	↓
oxidative stress and antioxidant status	plasma TEAC	1.19	1.00	1.07	1.13	1.15	1.18	↑
	plasma MDA	0.20	1.00	0.28	0.30	0.35	0.36	↓
	liver MDA	0.29	1.00	0.39	0.44	0.29	0.43	↓
	liver glutathione	3.13	1.00	2.60	2.54	2.85	2.83	↑

Values are expressed as fold change relative to the HFD-DW group (set as 1.0). Color intensity represents the magnitude of change, with red indicating a decrease and green indicating an increase relative to the HFD control. The direction of beneficial change for each parameter is indicated by arrows, facilitating interpretation based on physiological relevance. Parameters are grouped into glycemic control, lipid profile, and oxidative stress categories.

**Table 3:** Effects of enzymatic and non-enzymatic shallot extracts on organ weights and visceral adiposity in HFD-fed mice.

Parameters	NCD-DW	HFD-DW	HFD-NES1	HFD-NES2	HFD-ESE1	HFD-ESE2
Body weight (g)	33.50 ± 2.89	40.73 ± 3.62 *	40.63 ± 4.43 *	37.30 ± 2.10 *	37.89 ± 3.36 *	39.78 ± 2.78 *
Liver (g)	1.97 ± 0.21	1.89 ± 0.25 *	1.59 ± 0.33 *	1.52 ± 0.14 *^,^**	1.70 ± 0.27	1.93 ± 0.29
Right kidney (g)	0.25 ± 0.04	0.20 ± 0.03	0.20 ± 0.05	0.17 ± 0.01 *	0.18 ± 0.03 *	0.19 ± 0.02 *
Left kidney (g)	0.24 ± 0.03	0.20 ± 0.04	0.19 ± 0.03 *	0.18 ± 0.02 *	0.19 ± 0.03 *	0.18 ± 0.01 *
Heart (g)	0.18 ± 0.05	0.15 ± 0.01	0.16 ± 0.02	0.15 ± 0.02	0.16 ± 0.02	0.17 ± 0.01
Spleen (g)	0.09 ± 0.01	0.09 ± 0.01	0.10 ± 0.02	0.11 ± 0.01 *^,^**	0.11 ± 0.02 *^,^**	0.12 ± 0.01 *^,^**
Pancreas (g)	0.29 ± 0.05	0.37 ± 0.03	0.28 ± 0.09 **	0.28 ± 0.05 **	0.30 ± 0.09	0.36 ± 0.04
White adipose tissue (WAT)	0.18 ± 0.03	3.22 ± 0.79 *	2.77 ± 0.74 *	2.80 ± 0.46 *	2.84 ± 0.51 *	3.13 ± 0.69 *
Adiposity index *** (%WAT/BW)	0.53 ± 0.10	7.82 ± 1.44 *	6.76 ± 1.42 *	7.48 ± 1.02 *	7.47 ± 1.02 *	7.50 ± 1.19 *

Data are presented as mean ± SD. * *p* < 0.05 vs. NCD-DW; ** *p* < 0.05 vs. HFD-DW; Adiposity index was calculated as visceral white adipose tissue weight divided by final body weight × 100.

**Table 4:** Histopathological examination of major organs in HFD-fed mice treated with enzymatic and non-enzymatic shallot extracts.

Group	Liver	Kidney	Spleen	Heart	Pancreas
NCD-DW	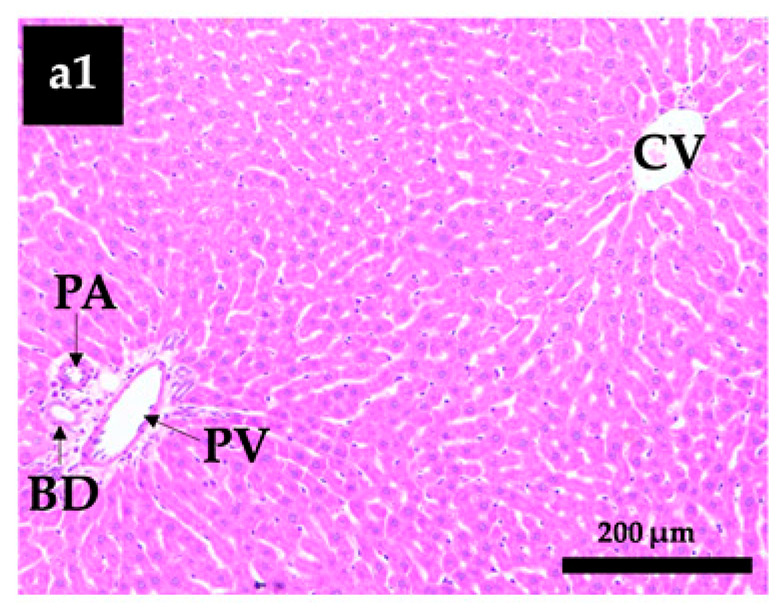	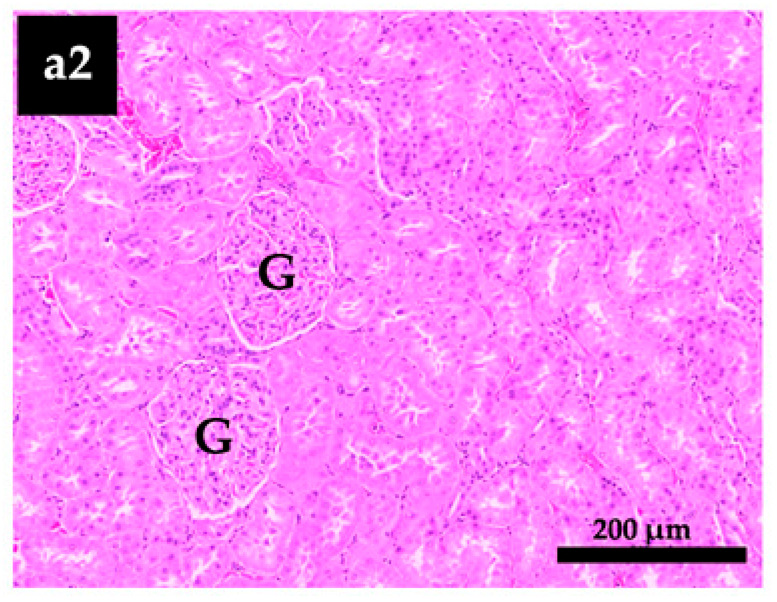	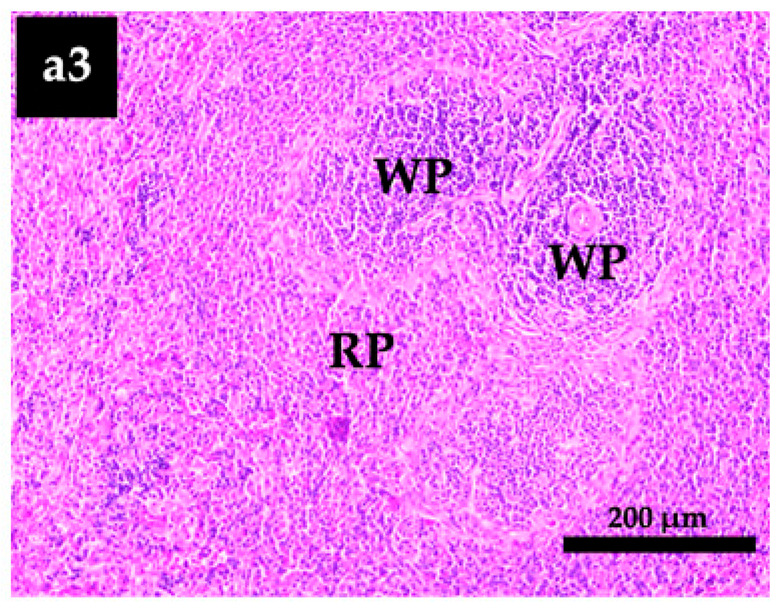	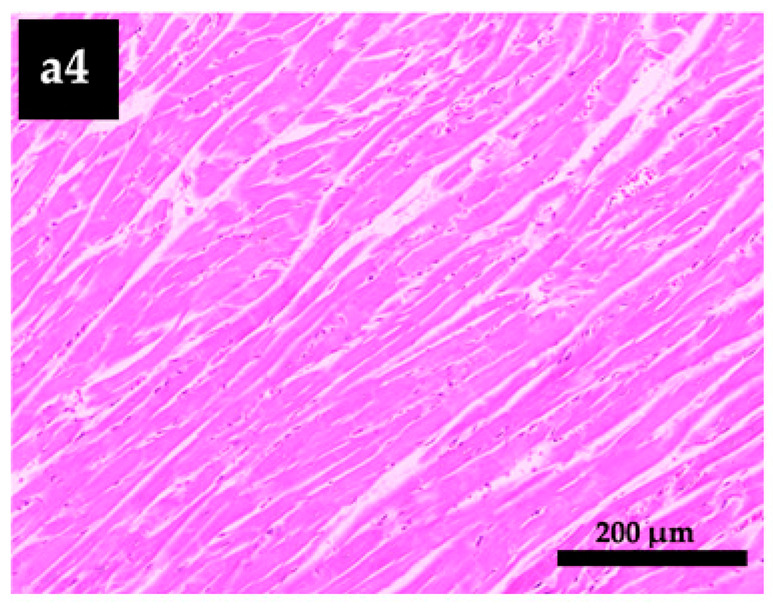	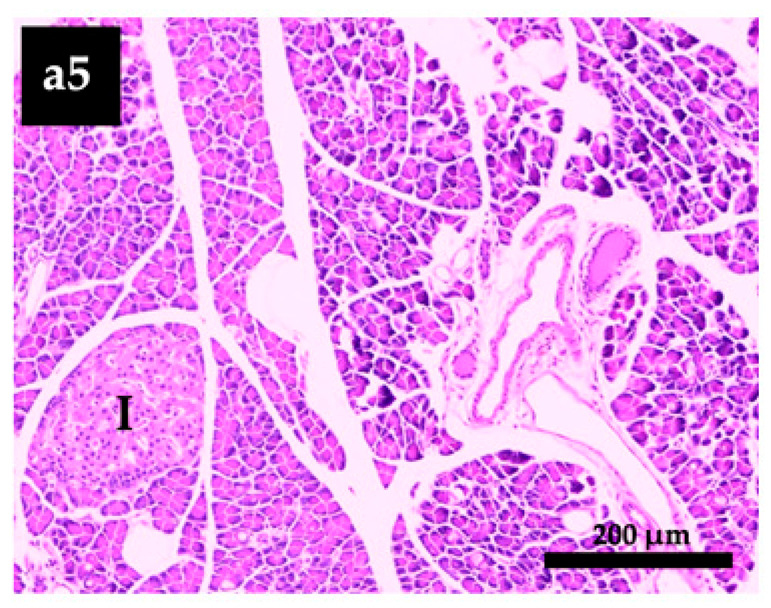
HFD-DW	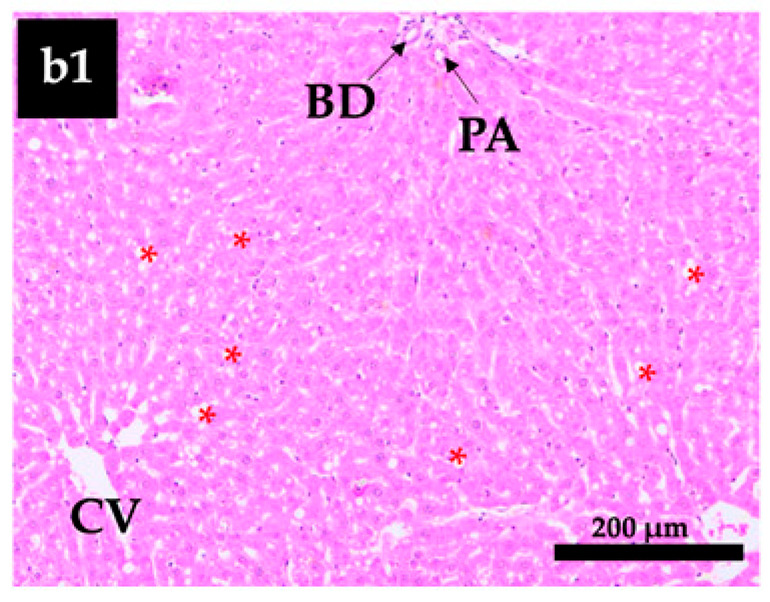	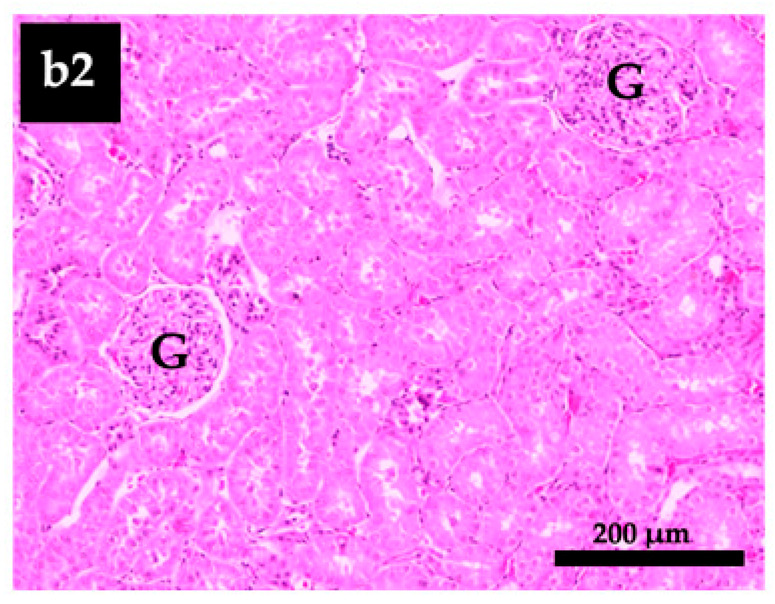	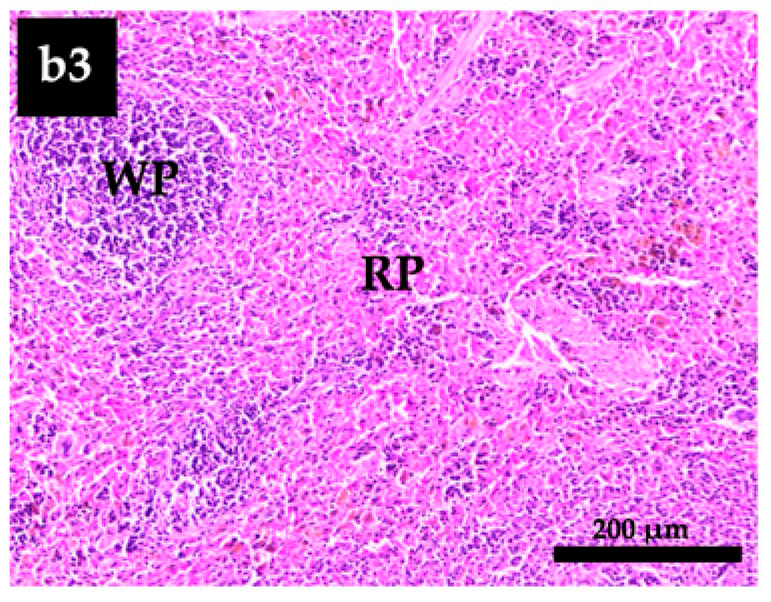	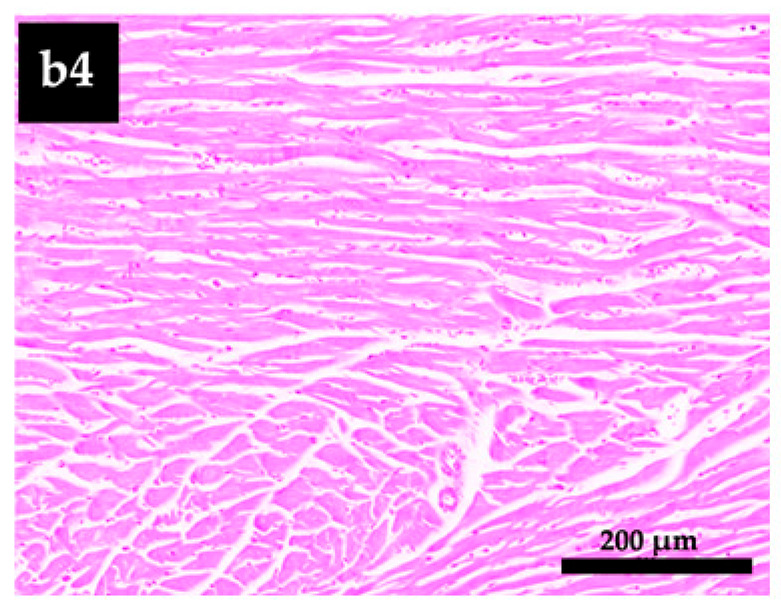	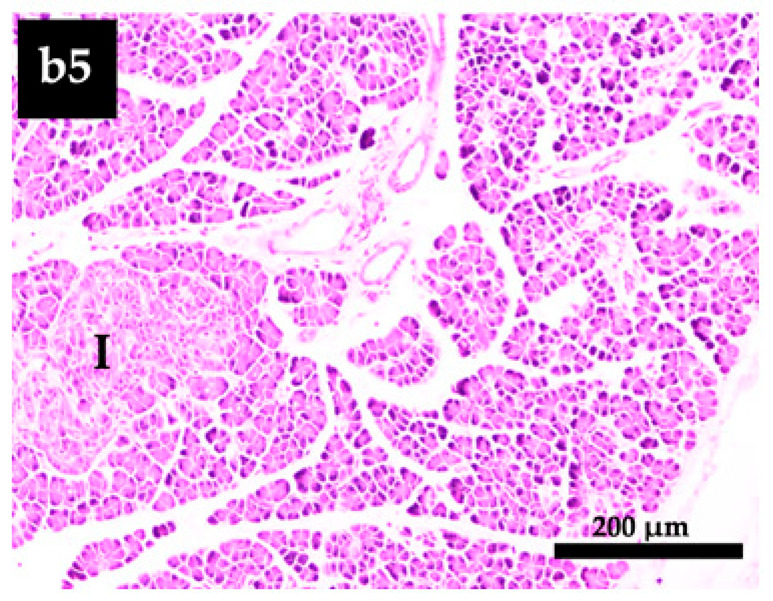
HFD-NES1	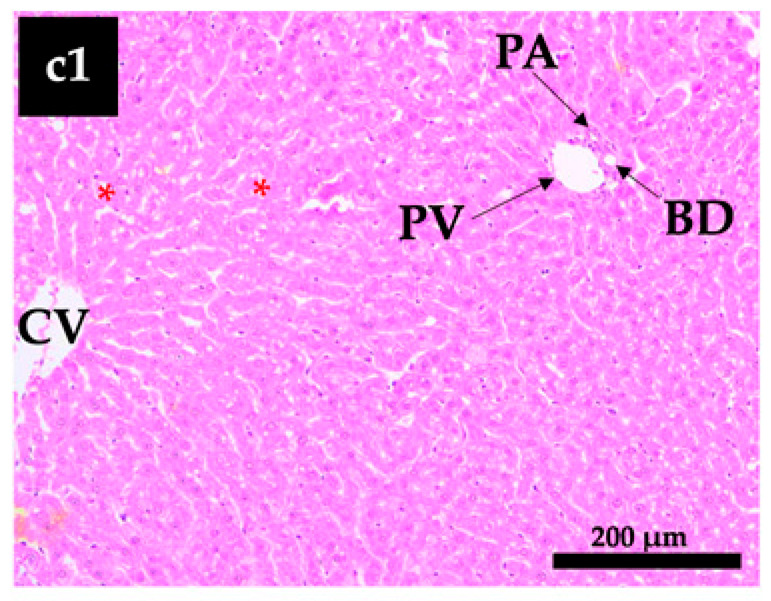	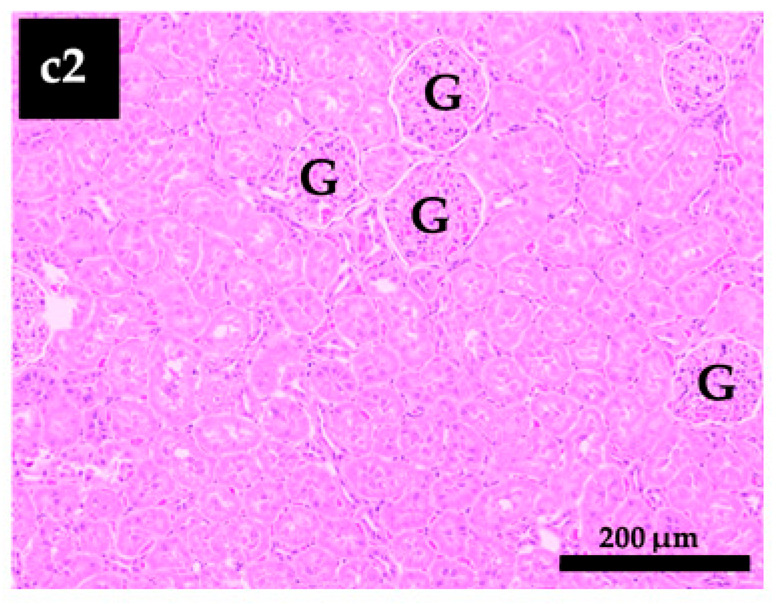	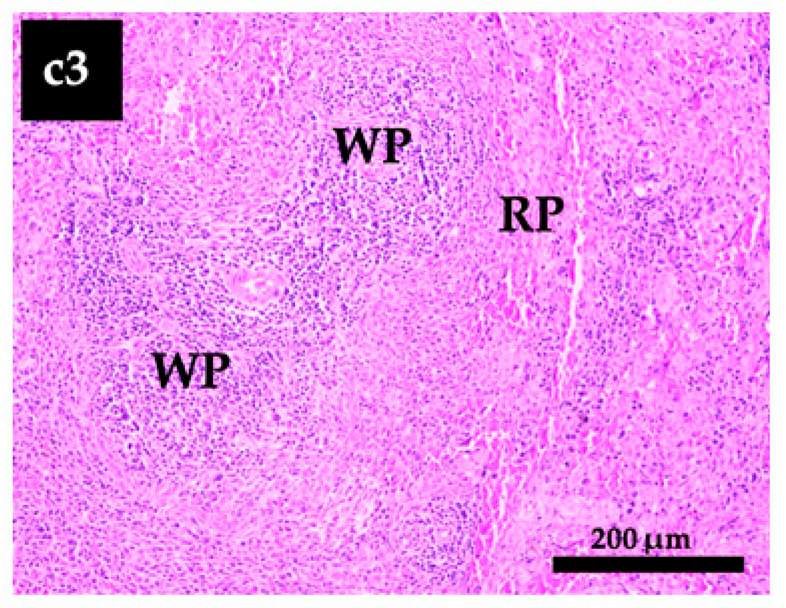	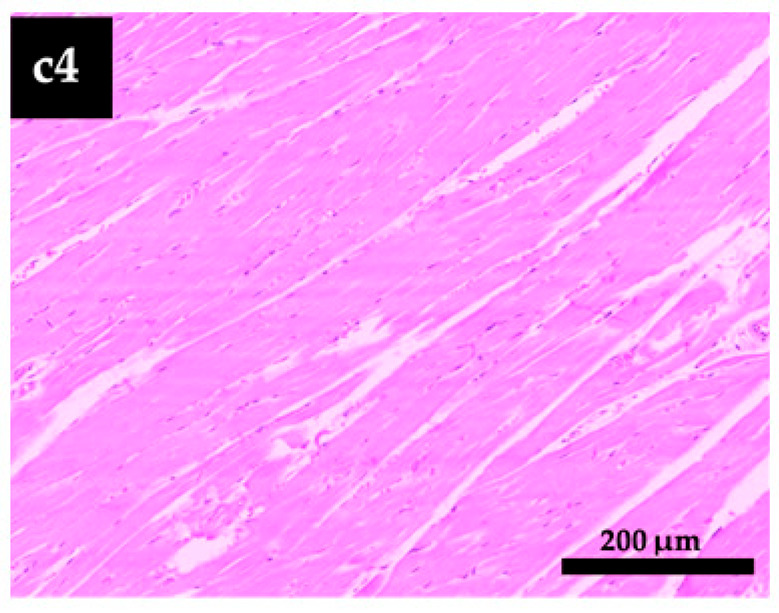	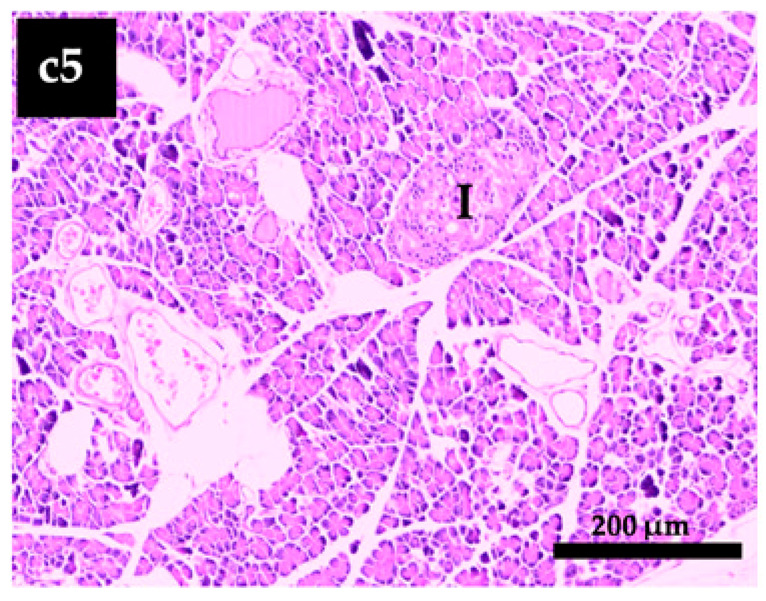
HFD-NES2	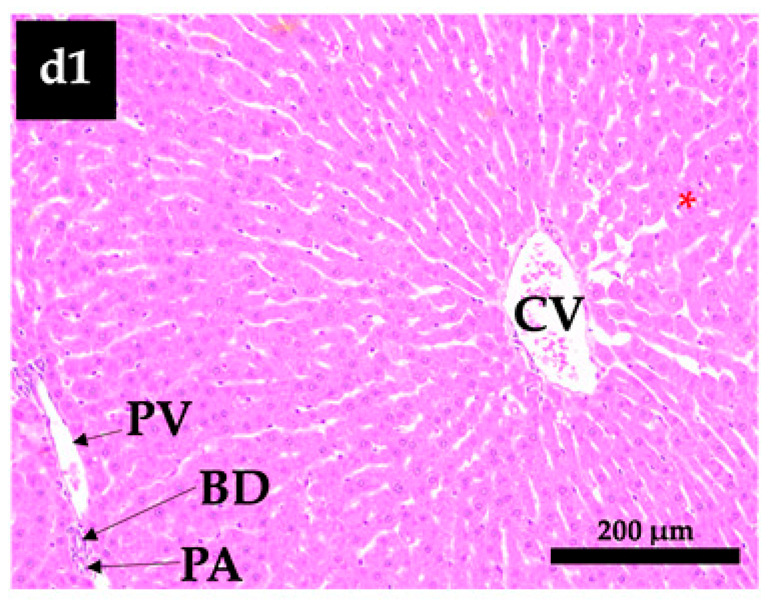	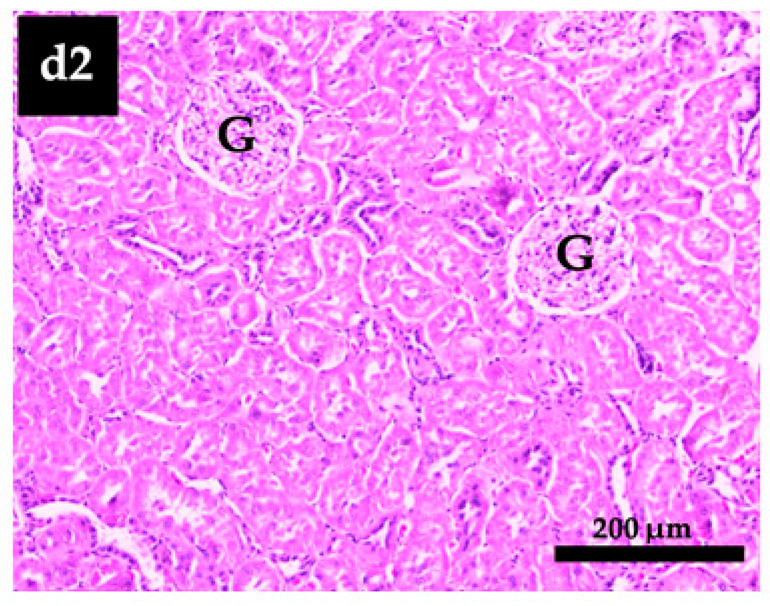	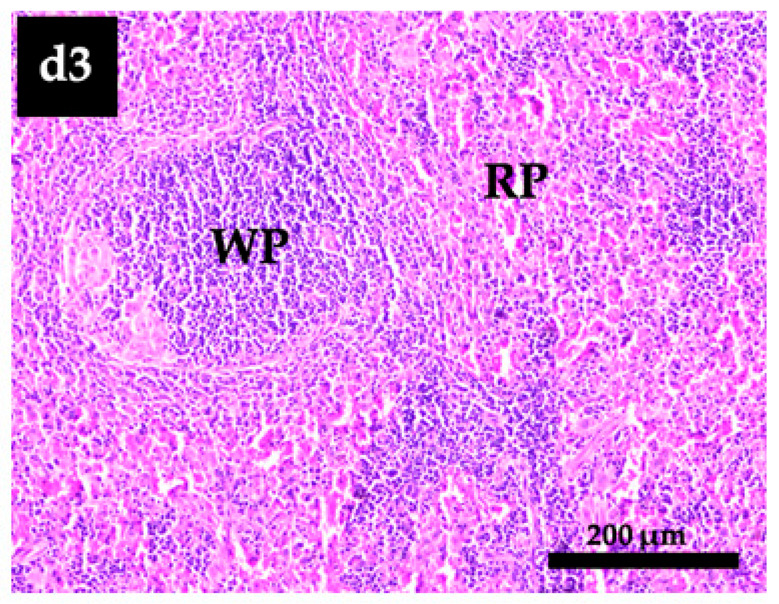	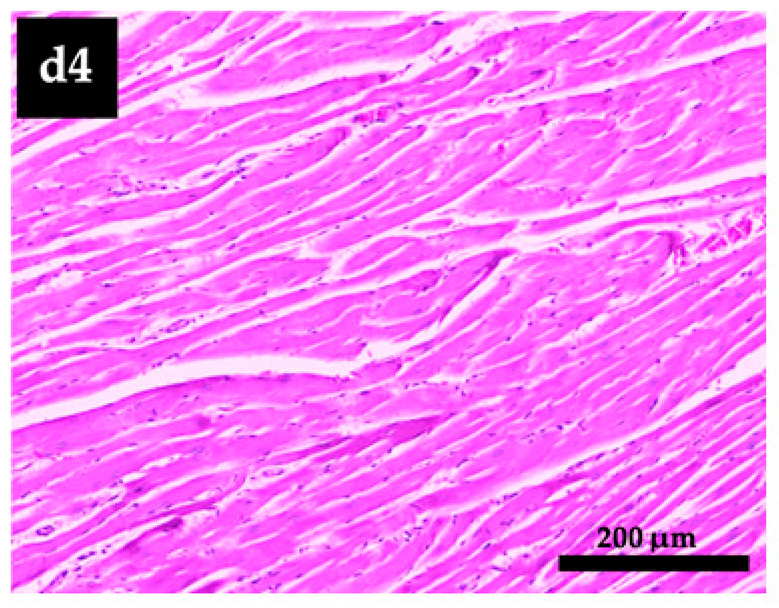	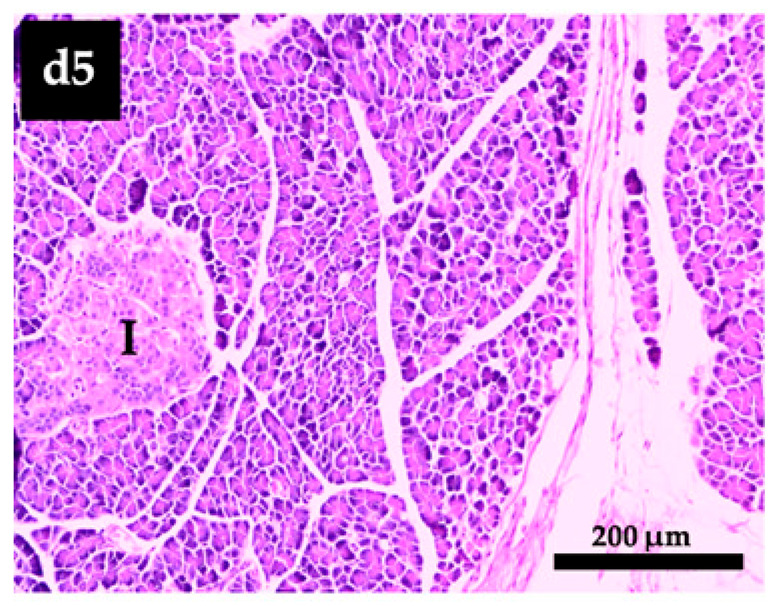
HFD-ESE1	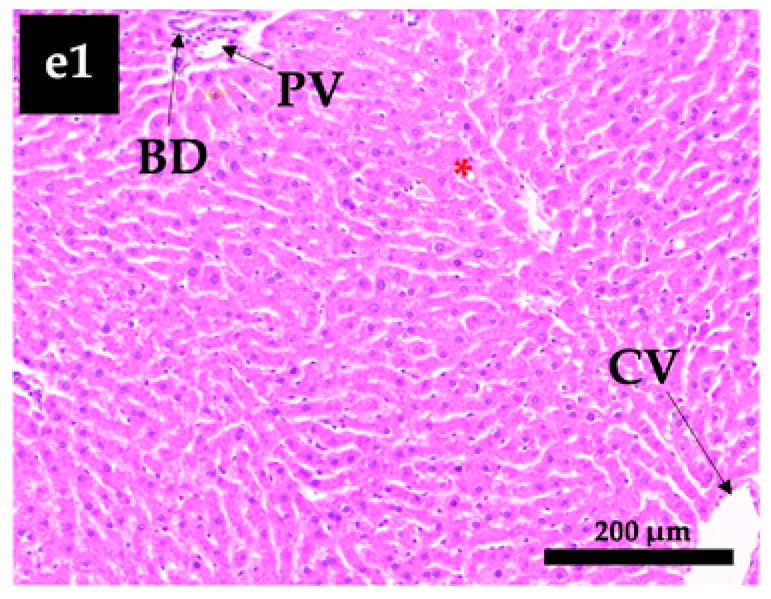	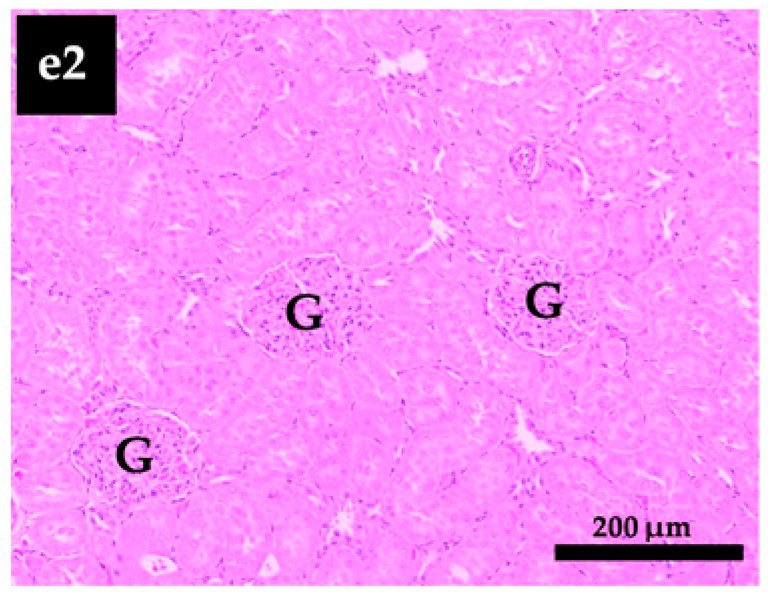	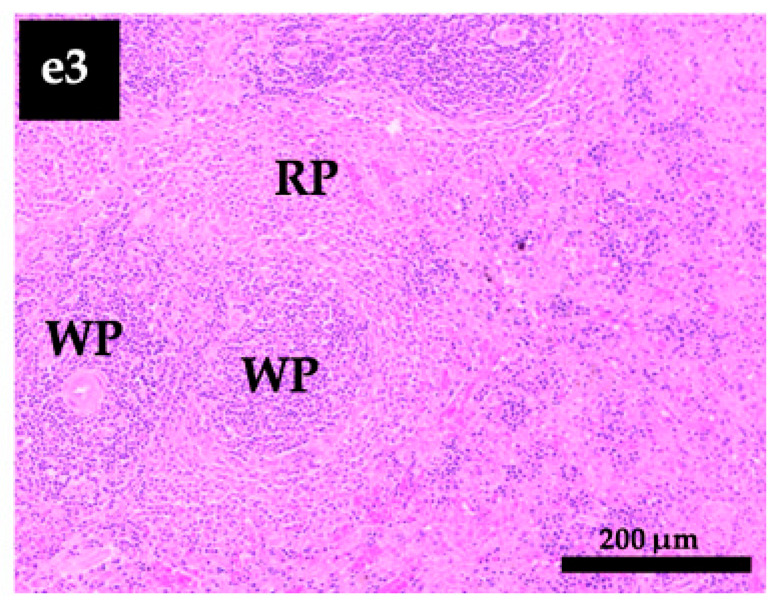	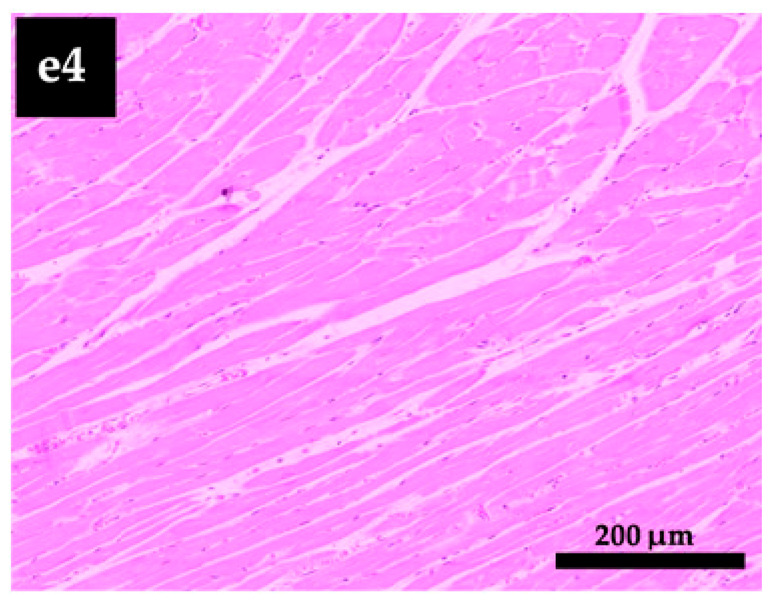	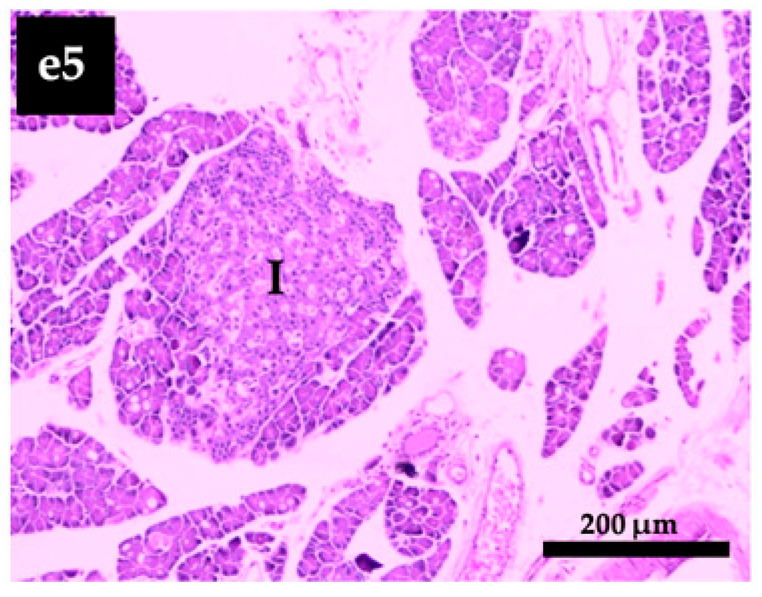
HFD-ESE2	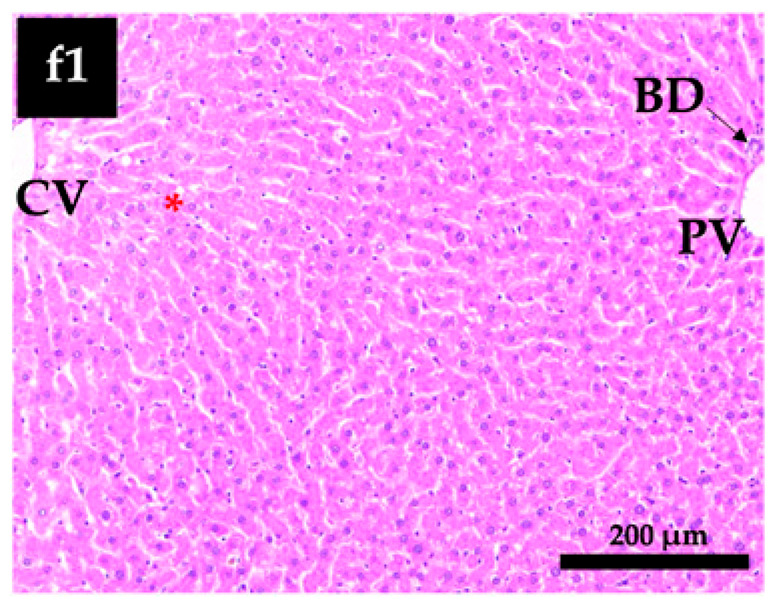	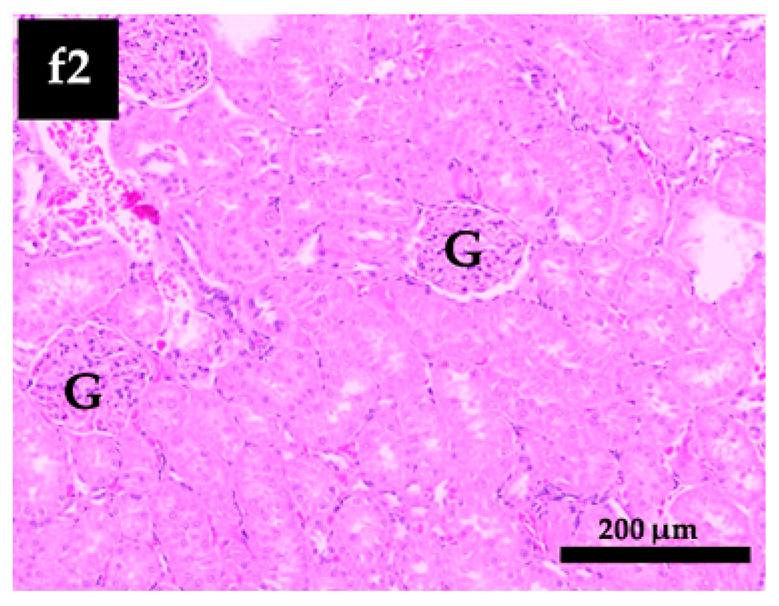	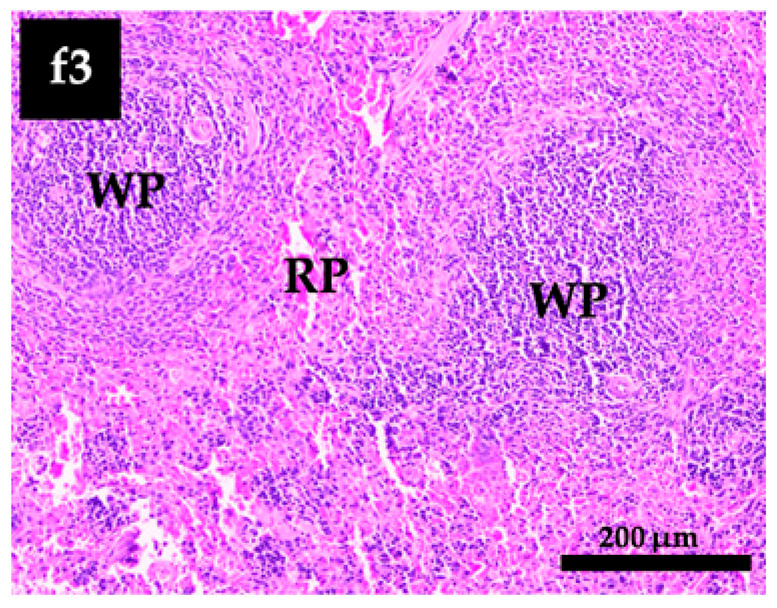	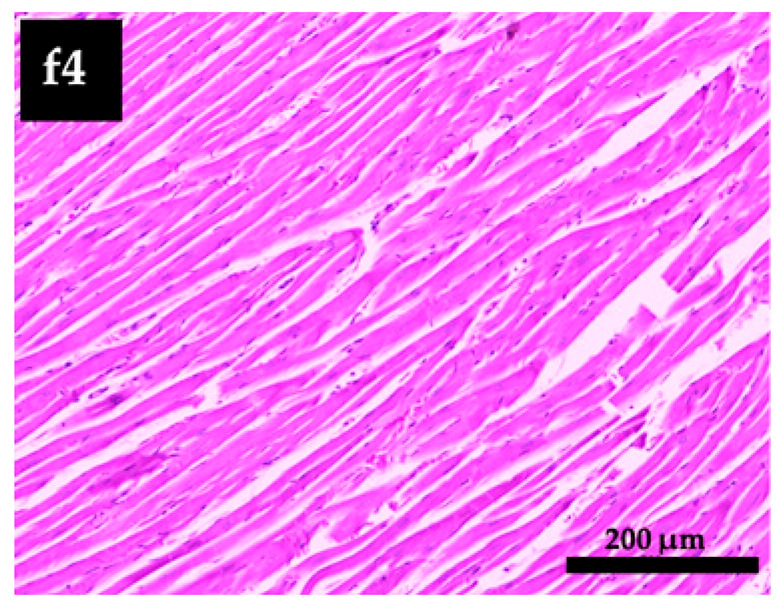	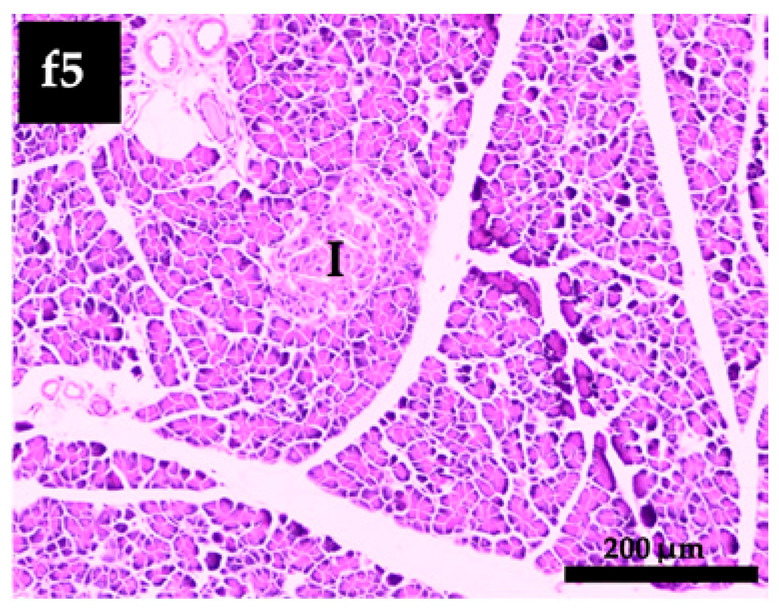

Representative histological sections of the liver, kidney, spleen, heart, and pancreas from mice fed a normal chow diet (NCD-DW), high-fat diet (HFD-DW), and HFD with shallot extract treatment (HFD-NES1, HFD-NES2, HFD-ESE1, and HFD-ESE2) following 12 weeks of intervention. Tissues were stained with hematoxylin and eosin (H&E) and examined under light microscopy. CV: central vein; PV: portal vein; PA: portal artery; BD: bile duct; *: lipid droplets; G: glomerulus; WP: white pulp; RP: red pulp; I: Islet of Langerhans.

## Data Availability

The authors declare that the data supporting the findings of this study are available within the article.

## Supplementary Materials

The following supporting information can be downloaded at: https://www.mdpi.com/article/10.3390/biology15130995/s1, Table S1: Weekly food intake of mice fed normal chow diet and high-fat diet during the 12-week induction period; Table S2: Estimated caloric intake of mice fed normal chow diet and high-fat diet during the 12-week induction period; Figure S1: Representative HPLC chromatograms of non-enzymatic and enzymatic Thai shallot extracts.
