# Supplementary material for: Comparable Metabolic and Histopathological Observations of Enzymatic and Non-Enzymatic Thai Shallot Extracts in High-Fat Diet-Induced Obese Mice
Source: Biology (Basel). 2026 Jun 24;15(13):995. doi: 10.3390/biology15130995 (PMC13360308; doi:10.3390/biology15130995)
Supplement: Supplementary file 1 [file biology-15-00995-s001.zip › biology-4364280-supplementary.pdf]

Supplementary Table S1. Weekly food intake of mice fed normal chow diet and high-fat diet during the 12-week induction period

Week	NCD food intake (g/mouse/day)	HFD food intake (g/mouse/day)	p-value
1	2.86 ± 0.28	2.78 ± 0.31	>0.05
2	2.91 ± 0.30	2.83 ± 0.27	>0.05
3	2.94 ± 0.25	2.88 ± 0.29	>0.05
4	3.01 ± 0.32	2.93 ± 0.34	>0.05
5	2.97 ± 0.27	2.90 ± 0.30	>0.05
6	3.05 ± 0.31	2.96 ± 0.28	>0.05
7	3.08 ± 0.29	3.00 ± 0.33	>0.05
8	3.12 ± 0.34	3.04 ± 0.30	>0.05
9	3.10 ± 0.28	3.02 ± 0.31	>0.05
10	3.15 ± 0.30	3.08 ± 0.35	>0.05
11	3.18 ± 0.33	3.11 ± 0.29	>0.05
12	3.20 ± 0.31	3.14 ± 0.32	>0.05

Data are presented as mean ± SD. NCD, normal chow diet; HFD, high-fat diet. No significant differences in food intake were observed between the NCD- and HFD-fed groups during the 12-week induction period.

Supplementary Table S2. Estimated caloric intake of mice fed normal chow diet and high-fat diet during the 12-week induction period

Week	NCD caloric intake (kcal/day)	HFD caloric intake (kcal/day)	p-value
1	10.90 ± 1.07	13.54 ± 1.51	<0.05
2	11.09 ± 1.14	13.78 ± 1.31	<0.05
3	11.20 ± 0.95	14.03 ± 1.41	<0.05
4	11.47 ± 1.22	14.27 ± 1.66	<0.05
5	11.32 ± 1.03	14.12 ± 1.46	<0.05
6	11.62 ± 1.18	14.42 ± 1.36	<0.05
7	11.74 ± 1.11	14.61 ± 1.61	<0.05
8	11.89 ± 1.30	14.80 ± 1.46	<0.05
9	11.81 ± 1.07	14.71 ± 1.51	<0.05
10	12.00 ± 1.14	15.00 ± 1.70	<0.05
11	12.12 ± 1.26	15.15 ± 1.41	<0.05
12	12.19 ± 1.18	15.29 ± 1.56	<0.05

Data are presented as mean ± SD. Caloric intake was calculated by multiplying daily food intake by the energy density of each diet (NCD = 3.811 kcal/g; HFD = 4.870 kcal/g).

Although daily food intake (g/day) did not differ significantly between NCD- and HFD-fed mice, estimated caloric intake was consistently higher in the HFD group because of the greater energy density of the high-fat diet (4.870 kcal/g vs. 3.811 kcal/g).

Supplementary Figure S1. Representative HPLC chromatograms of non-enzymatic and enzymatic Thai shallot extracts.

(A) Representative HPLC chromatogram of non-enzymatic shallot extract (NES).

(B) Representative HPLC chromatogram of enzymatic shallot extract (ESE) following β-glucosidase treatment.

Chromatographic separation was performed using a Shimadzu LC-20A HPLC system equipped with a diode-array detector, and detection was monitored at 360 nm. Enzymatic hydrolysis markedly altered the flavonoid profile of the extract, resulting in a substantial increase in quercetin aglycone and a corresponding decrease in quercetin glycosides compared with the non-enzymatic extract. Peak identities were assigned by comparison of retention times and UV–Vis spectra with authentic standards and were used for quantitative analysis presented in Table 1.

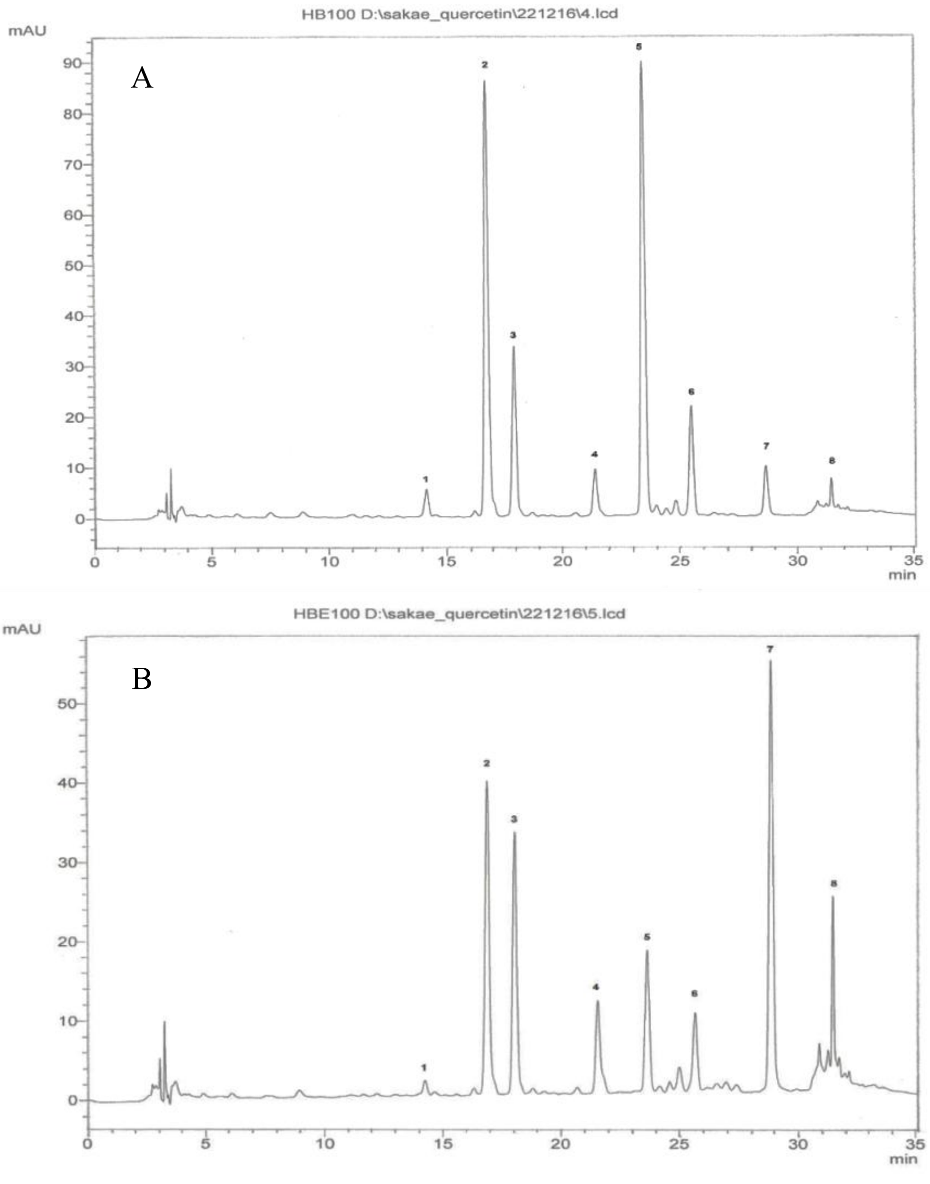

Shallot extract chromatogram (λ=360). Peak: (2) Quercetin-3,4’-diglucoside (4) Quercetin-3-glucoside (5) Quercetin-4’-glucoside (7) Quercetin and (1,3,4,6) non-identified quercetin glycoside
